# Essential role for centromeric factors following p53 loss and oncogenic transformation

**DOI:** 10.1101/gad.290924.116

**Published:** 2017-03-01

**Authors:** Dan Filipescu, Monica Naughtin, Katrina Podsypanina, Vincent Lejour, Laurence Wilson, Zachary A. Gurard-Levin, Guillermo A. Orsi, Iva Simeonova, Eleonore Toufektchan, Laura D. Attardi, Franck Toledo, Geneviève Almouzni

**Affiliations:** 1 Institut Curie, Paris Sciences et Lettres (PSL) Research University, UMR3664, Centre Nationnal de la Recherche Scientifique (CNRS), Equipe Labellisée Ligue contre le Cancer, F-75005 Paris, France;; 2 Sorbonne Universités, Université Pierre et Marie Curie (UPMC) Université Paris 06, UMR3664, CNRS, F-75005 Paris, France;; 3 Institut Curie, PSL Research University, UMR3244, CNRS, Equipe Labellisée Ligue contre le Cancer, F-75005 Paris, France;; 4 Sorbonne Universités, UPMC Université Paris 06, UMR3244, CNRS, F-75005 Paris, France;; 5 Division of Radiation and Cancer Biology, Department of Radiation Oncology, Stanford University School of Medicine, Stanford, California 94305, USA;; 6 Department of Genetics, Stanford University School of Medicine, Stanford, California 94305, USA

**Keywords:** HJURP, CENP-A, p53, oncogenic transformation, centromere

## Abstract

In mammals, centromere definition involves the histone variant CENP-A (centromere protein A), deposited by its chaperone, HJURP (Holliday junction recognition protein). Alterations in this process impair chromosome segregation and genome stability, which are also compromised by p53 inactivation in cancer. Here we found that CENP-A and HJURP are transcriptionally up-regulated in p53-null human tumors. Using an established mouse embryonic fibroblast (MEF) model combining p53 inactivation with E1A or HRas-V12 oncogene expression, we reproduced a similar up-regulation of HJURP and CENP-A. We delineate functional CDE/CHR motifs within the *Hjurp* and *Cenpa* promoters and demonstrate their roles in p53-mediated repression. To assess the importance of HJURP up-regulation in transformed murine and human cells, we used a CRISPR/Cas9 approach. Remarkably, depletion of HJURP leads to distinct outcomes depending on their p53 status. Functional p53 elicits a cell cycle arrest response, whereas, in p53-null transformed cells, the absence of arrest enables the loss of HJURP to induce severe aneuploidy and, ultimately, apoptotic cell death. We thus tested the impact of HJURP depletion in pre-established allograft tumors in mice and revealed a major block of tumor progression in vivo. We discuss a model in which an “epigenetic addiction” to the HJURP chaperone represents an Achilles’ heel in p53-deficient transformed cells.

## Abstract (precis)

In this study, Filipescu et al. find that CENP-A and HJURP, a CENP-A chaperone, are transcriptionally up-regulated in p53-null human tumors. They tested the impact of HJURP depletion in pre-established allograft tumors in mice and revealed a major block of tumor progression in vivo, leading them to propose an “epigenetic addiction” model in which HJURP chaperone represents an Achilles’ heel in p53-deficient transformed cells.

Centromeres mark the site of kinetochore formation, ensuring equal distribution of the two sets of chromosomes during mitosis (Allshire and Karpen 2008; Black et al. 2010). Their identity is defined epigenetically by the histone H3 variant CENP-A (centromere protein A in mammals, also designated as CenH3) (Earnshaw and Rothfield 1985; Van Hooser et al. 2001; Talbert et al. 2012). The mechanism of cell cycle-dependent CENP-A deposition at centromeres in different model systems is now well described (for review, see Müller and Almouzni 2014, 2017; Kinley and Cheeseman 2016). In mammals, CENP-A is deposited at centromeres in early G1 phase (Jansen et al. 2007) by its dedicated histone chaperone, HJURP (Holliday junction recognition protein) (Dunleavy et al. 2009; Foltz et al. 2009; Shuaib et al. 2010). HJURP localization and licensing to incorporate CENP-A at centromeres are linked to cell cycle progression through Cdk (cyclin-dependent kinase) activity and interaction with cyclin A (Silva et al. 2012; Stankovic et al. 2017). Importantly, centromere deregulation can lead to genome instability, a hallmark of cancer (Tanaka and Hirota 2009; Thompson et al. 2010). CENP-A knockout in mice shows severe mitotic defects (Howman et al. 2000), which arise through the impaired recruitment of downstream centromere components as well as the failure of the kinetochore to correctly assemble at the centromere and ensure faithful chromosome segregation (Régnier et al. 2005; Fachinetti et al. 2013; Hoffmann et al. 2016). Remarkably, HJURP ablation in cell lines also causes mitotic defects due to the loss of CENP-A at centromeres (Dunleavy et al. 2009; Foltz et al. 2009). These latter findings underscore a key role for the HJURP chaperone in centromere function.

CENP-A overexpression has been reported in several human cancers, including breast (Ma et al. 2003; Montes de Oca et al. 2015), colorectal (Tomonaga et al. 2003), liver (Li et al. 2011), lung (Wu et al. 2012), ovarian (Qiu et al. 2013), and osteosarcoma (Gu et al. 2014). HJURP overexpression was first identified in lung (Kato et al. 2007) and further observed in breast (Hu et al. 2010; Montes de Oca et al. 2015), glioma (de Tayrac et al. 2013), and astrocytoma (Valente et al. 2013). Furthermore, in breast cancer, elevated HJURP levels emerged as an independent prognostic marker of poor patient outcome, distinguishing aggressive tumors within the luminal A subtype (Montes de Oca et al. 2015). However, to date, the causal relationship between aberrant expression of these factors and cancer development has not yet been established. It is equally unknown whether this up-regulation is limited to particular types of cancer or at what point it occurs during tumor evolution. Initial work in human cancer cell lines focusing on exogenously overexpressed CENP-A (Lacoste et al. 2014) showed that it can mislocalize outside the centromere in euchromatin, which is equally the case for the endogenously up-regulated protein (Athwal et al. 2015). Given how H3.1 and H3.3 oncohistones harboring K-to-M mutations contribute to cell type-specific tumors such as glioblastomas, chondroblastomas, and sarcomas (Schwartzentruber et al. 2012; Sturm et al. 2012; Fang et al. 2016; Lu et al. 2016), a tempting hypothesis could be that aberrant CENP-A expression might drive tumorigenesis in a cell type-specific manner.

In addition, intriguing connections have been made between the tumor suppressor p53 and several chromatin regulators. p53 typically functions as a transcriptional activator to induce anti-proliferative responses to cellular stresses such as DNA damage, genome instability, hypoxia, or oncogenic signaling and is mutated in at least half of human cancers (for review, see Bieging et al. 2014). This role is in line with missense gain-of-function mutations in p53, which lead to aberrant transcriptional activation of chromatin regulators such as MLL2, resulting in elevated histone methylation and acetylation genome-wide, favoring cancer development (Zhu et al. 2015). In addition to activation, p53 can also repress the expression of particular genes, such as a subset required for DNA repair and telomere maintenance (Simeonova et al. 2013; Jaber et al. 2016). The impact of p53-dependent transcriptional regulation on centromeric factors is thus important to consider. Indeed, p53 is known to sense chromosomal breaks and defects induced by mitotic dysfunction and respond by promoting cell cycle arrest to prevent genome instability (Harvey et al. 1993; Murphy and Rosen 2000; Ghiselli 2006; Kim et al. 2009; Lambrus et al. 2015; Ohashi et al. 2015) Such defects comprise aneuploidy (defined as hyperploid and hypoploid chromosome numbers), which is a frequent outcome of aberrant mitosis and gives rise to genome instability, a hallmark of cancer (Santaguida and Amon 2015). In cells with functional p53, defects in chromosome segregation activate p53 by several mechanisms, mediated in part by ATM or p38 (Santaguida and Amon 2015). Interestingly, HJURP and CENP-A are among the multiple factors whose expression is regulated by p53 in response to aneuploidy induced by pharmacological inhibition of Aurora kinases (Li et al. 2009). In nontransformed primary human fibroblasts, depletion of either the chaperone or the centromeric histone variant leads to a p53-induced cellular senescence response to protect cells from performing aberrant mitoses (Maehara et al. 2010; Heo et al. 2013). However, the role of p53 in connection to HJURP and CENP-A overexpression in cancer cells has not been explored to date.

Based on these connections, we searched for specific types of human cancers in which CENP-A and HJURP mRNA is up-regulated. Interestingly, we found a significant increase in expression of these chromatin factors in tumors with *TP53*-inactivating mutations relative to tumors with intact *TP53*. We further identified that p53 represses the murine *Hjurp* and *Cenpa* genes through the functional CDE/CHR motifs in their promoter regions, providing a direct mechanism for the control of their expression. Thus, loss of p53 unleashes expression of two key factors for centromere definition. We thus wanted to determine how CENP-A and, more specifically, HJURP overexpression could contribute to tumorigenesis. First, we used a primary mouse embryonic fibroblast (MEF) model in which the loss of p53 acts as a defined “first hit,” and a “second hit” caused by expressing one or more oncogenes together can induce cellular transformation. We found that both HJURP and CENP-A became up-regulated following p53 loss and even further following oncogenic transformation, as in the data from tumor samples. Thus, we could exploit this system to dissect the role of HJURP and CENP-A overexpression in p53-null cells in comparison with cells with functional p53. Our data led us to propose a model for “epigenetic addiction” in which the rapidly proliferating cells in p53-null tumors become highly dependent on the HJURP chaperone.

## Results

### CENP-A and HJURP are overexpressed in p53-null human tumors

**Figure 1.**
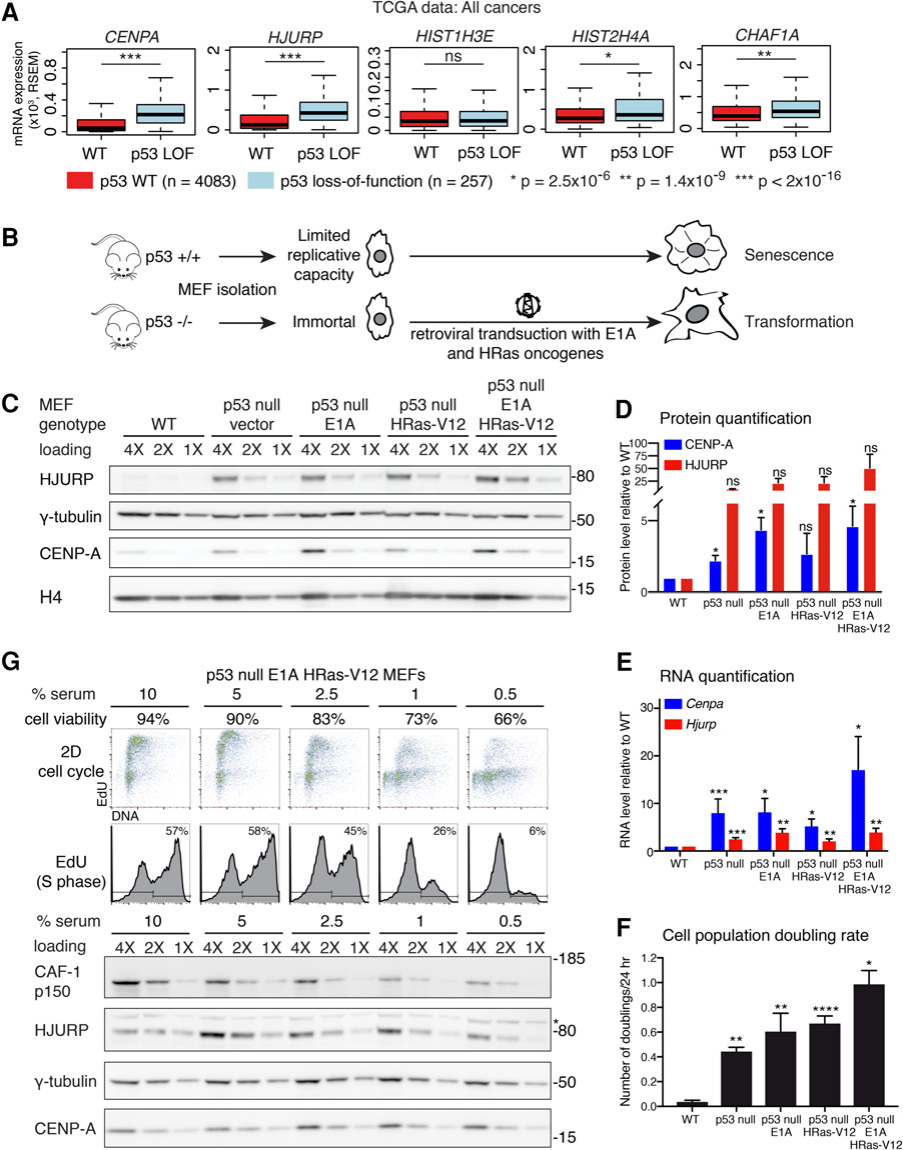
CENP-A and HJURP levels are increased in human tumors and MEFs with inactivated p53. (*A*) Box plot comparisons of relative expression (mRNA) of genes coding for CENP-A (*CENPA*), HJURP (*HJURP*), CAF-1 p150 (*CHAF1A*), H3.1 (*HIST1H3E*), and H4 (*HIST2H4A*) from all cancers (28 cancer types), classified according to p53 status (TCGA provisional data). Tumors are either wild type for *TP53* (diploid with no detectable mutations; *n* = 4083) or p53 loss of function (LOF) (homozygous deletion or heterozygous deletion + *TP53* mutation featuring nonsense or in-frame truncations, resulting in p53 inactivation; *n* = 257). All other *TP53* mutants were excluded. mRNA levels are expressed in RSEM (RNA-seq by expectation maximization) units. We used Wilcoxon rank sum tests to compute significance. (*B*) Experimental scheme outlining the MEF cellular system used in this study. MEFs extracted from wild-type mice have a limited replicative capacity in cell culture. Mice lacking p53 (p53-null) are immortal and can be transformed by a single oncogene, which we introduced into cells by retroviral transduction of E1A and HRas-V12, expressed under the control of the viral promoter. (*C*) Western blot of HJURP and CENP-A levels in RIPA-soluble extracts from wild-type MEFs or p53-null MEFs transduced with empty vector, E1A, or HRas-V12 or sequentially with E1A and HRas-V12. γ-Tubulin was used as a loading control. H4 levels are also shown. A twofold dilution series of each extract is represented by 4X, 2X, and 1X. Molecular weight protein markers are indicated at the *right*. (*D*) Quantification of HJURP and CENP-A protein levels from the MEFs described in *C*. Results are represented as fold change compared with wild-type cells. Error bars represent the SEM of three experiments. (*E*) RT-qPCR analysis of *Cenpa* and *Hjurp* mRNA levels in the MEFs described in *C*. Results are represented as fold change compared with wild-type cells. Error bars represent the SEM of at least three experiments. (*F*) Proliferation rate is represented as the average of cell doublings per 24 h for the MEFs described in *C*. MEFs (3 × 10^4^) were seeded in 1 mL of growth medium on 24-well plates. Error bars represent the SEM from triplicate experiments. (*D*–*F*) (****) *P* < 0.0001; (***) *P* ≤ 0.0005; (**) *P* ≤ 0.005; (*) *P* ≤ 0.05; (NS) not significant, significance determined by a *t*-test. (*G*) Cell cycle and Western blot analysis of p53-null, E1A, and HRas transformed MEFs grown in medium containing decreasing concentrations of serum (10%–0.5%, as indicated) for 96 h to decrease their proliferative rate. We determined cell viability by trypan blue exclusion (percentage of viable cells indicated). (*Top* panels) Cell cycle analysis by flow cytometry, performed following EdU and propidium iodide (PI) staining. The percentage of EdU-positive cells (S phase) is indicated. Western blot of HJURP and CENP-A levels in RIPA-soluble extracts. The CAF-1 p150 subunit was used as a proliferation marker (Polo et al. 2010), and γ-tubulin was used as a loading control. A twofold dilution series of each extract is represented by 4X, 2X, and 1X. Molecular weight protein markers are indicated at the *right*.

In order to identify the specific context in which HJURP and CENP-A are transcriptionally up-regulated in human cancers, we first explored data from The Cancer Genome Atlas (TCGA). We grouped human tumors according to *TP53* status: wild-type p53 (diploid with no detectable mutations) and p53 loss of function (mutations leading to p53 inactivation, such as p53 homozygous deletion or heterozygous deletion, and a nonsense mutation or in-frame truncation of the second allele). All other heterozygous p53 mutations were excluded. We observed an increase in *HJURP* and *CENPA* RNA levels in several distinct p53 loss-of-function cancers, including breast cancer, melanoma, and pancreatic cancer (Supplemental Fig. S1A). The trend remains the same across various tumors, although the increase is not always statistically significant, presumably due to small sample size. We thus pooled 28 available cancer types of different cellular origin and found that *HJURP* and *CENPA* expression is increased in tumors with p53-inactivating mutations (*P* < 2 × 10^−16^) (Fig. 1A). Thus, this increase is not specific to a particular tumor type but rather relates to the p53-deficient status of the tumors. Importantly, the expression of the replicative histone variant H3.1 gene is not increased, indicating that this is not a general regulatory mechanism affecting histone H3 variants indiscriminately. Histone H4 shows a slight increase in p53 mutant tumors (*P* = 2.5 × 10^−6^) (Fig. 1A), consistent with a necessary coregulation, considering its ability to form dimers with CENP-A. Notably, the p53 mutant tumors feature increased expression of the large subunit of the CAF-1 complex p150 (*CHAF1A*), which has been shown to correlate with increased proliferation (Polo et al. 2004). This suggests that the loss of p53 could lead to the abnormal accumulation of these centromeric factors during cancer progression.

### HJURP and CENP-A levels increase in p53-null MEFs following oncogenic transformation: cause or consequence?

We next wanted to test whether HJURP and CENP-A up-regulation is a cause or a consequence of cellular transformation in a p53-null context. Given the diverse histological origin of p53-null human tumors with HJURP and CENP-A overexpression, we selected a cellular model system of broad significance for transformation that is classically used to discover oncogene and tumor suppressor function rather than a model for a specific malignancy. MEFs wild-type for p53 have a limited growth potential due to p53-mediated onset of premature senescence (Lowe et al. 1994; Serrano et al. 1997). In contrast, p53-null MEFs are immortal and can be transformed with a single oncogene such as constitutively active HRas (HRas-V12) or the adenoviral E1A oncoprotein (Fig. 1B). Therefore, we chose primary MEF cells as a model system to study the contribution of HJURP and CENP-A to oncogenic transformation in a broad tumor context in connection to p53. We first transduced p53-null MEFs with retroviruses encoding the E1A adenovirus oncoprotein, the HRas-V12 oncogenic mutant, or both E1A and HRas-V12. While we could barely detect endogenous HJURP or CENP-A in wild-type MEFs, their levels increased in immortal p53-null MEFs and reached their highest point in cells further transformed with E1A, HRas-V12, or both oncogenes (Fig. 1C,D). This increase at the protein level for both HJURP and CENP-A was paralleled at the RNA level as shown by RT-qPCR (Fig. 1E) and also correlated with the increased proliferation rate of the cells (Fig. 1F).

The association of p53 loss with HJURP and CENP-A up-regulation in both human tumors and the primary MEF cellular system strongly suggested p53-mediated repression of both the *Hjurp* and *Cenpa* genes. However, we first wanted to exclude the possibility that the further increase in HJURP and CENP-A levels observed upon oncogenic transformation of MEFs could simply reflect an increased proliferation rate. Since, in human cells, *CENPA* expression and translation peak in the G2 phase (Shelby et al. 1997, 2000), we first verified whether a similar cell cycle regulation of *Cenpa* and *Hjurp* was occurring in mouse cells. We used the NIH/3T3 immortalized fibroblast line, whose stable chromosome number (Leibiger et al. 2013), in contrast to p53-null MEFs, allows the sorting of an unperturbed asynchronous population into cell cycle stages (Supplemental Fig. S1C). Similar to human cells, RT-qPCR analysis on sorted NIH/3T3 cells revealed increased *Cenpa* and *Hjurp* expression in S and G2 phases compared with G1 (Supplemental Fig. S1D). If indeed the up-regulation of HJURP and CENP-A was simply reflecting an increased proportion of S and G2 during hyperproliferation, one would expect that their levels would decrease upon a reduction in the proliferation rate. To address this possibility, we cultured p53-null E1A/HRas-V12 transformed MEFs in medium containing decreasing concentrations of serum for 96 h. Serum starvation caused a dose-dependent decrease in the proportion of cells in S phase and an increase in cell death. This was reflected at the protein level with the p150 subunit of the CAF-1 complex, which displayed a dose-dependent reduction (Fig. 1G), consistent with its role in histone deposition coupled to DNA replication during S phase (Kaufman et al. 1995) and with its previous characterization as a marker of proliferation (Polo et al. 2004). In contrast, CENP-A and HJURP levels remained stable with serum starvation (Fig. 1G). This suggests that the up-regulation of HJURP and CENP-A does not simply reflect increased proliferation but rather that these genes could be repressed by p53 and that this repression is lost in cancers following p53 inactivation.

### Identification of functional p53 regulatory elements in the *Hjurp* and *Cenpa* genes

**Figure 2.**
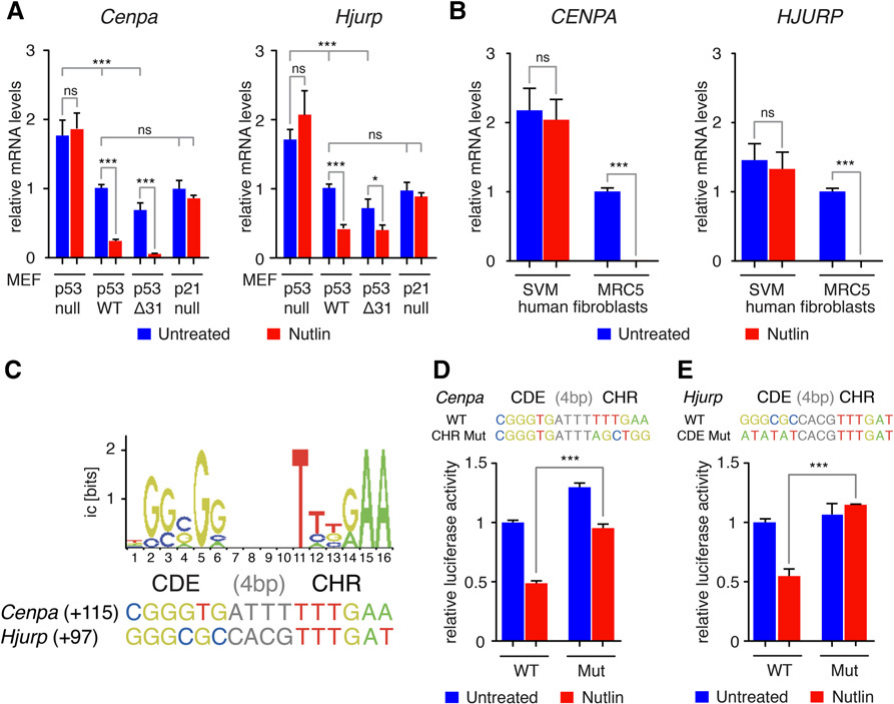
Cenpa and Hjurp are down-regulated upon p53 activation. (*A*) RT-qPCR analysis of CENP-A and HJURP mRNA levels in p53-null, wild-type (WT), p53^Δ31/Δ31^ (Δ31), or p21-null MEFs untreated or treated with 10 µM nutlin for 24 h. Results from three (or two for p21-null) independent experiments are shown, each quantified in triplicate. (*B*) RT-qPCR analysis of CENP-A and HJURP mRNA levels in normal human fibroblasts (MRC5) or their SV40 transformed derivative cells (SVM for SV40 MRC5) in which p53 is inactive untreated or treated with 10 µM nutlin for 24 h. Results from three experiments are shown, each quantified in triplicate. (*C*) Putative CDE/CHR motifs identified near the transcription start sites (TSSs) of *Cenpa* and *Hjurp* using the positional frequency matrix (shown at the *top*; for details see Supplemental Fig. 2). Candidate CDE/CHR motifs were found close to the TSS of each gene; numbers in parentheses are positions relative to TSS. For *Cenpa* the CHR element perfectly matches the consensus sequence, whereas the CDE element perfectly matches the consensus sequence for *Hjurp*. (*D*,*E*) A 2-kb-long fragment centered around the *Hjurp* and *Cenpa* TSS, respectively, containing a wild-type or mutant CHR motif (for *Cenpa*) or CDE motif (for *Hjurp*) was cloned upstream of a luciferase gene and transfected into NIH/3T3 cells. The putative CDE/CHR and its mutated counterpart are shown. Nutlin treatment for 24 h induces a strong decrease in luciferase activity only with the construct containing a wild-type CDE/CHR motif. Data from two independent experiments (each in duplicates) were normalized, and the average luciferase activity in untreated cells transfected with the wild-type promoter construct was assigned a value of 1. For all graphs, means + SEM are shown. (***) *P* ≤ 0.001; (*) *P* ≤ 0.05; (NS) not significant, by ANOVA or Student's *t*-tests.

To test for a putative p53-mediated down-regulation of *Cenpa* and *Hjurp*, we compared mRNA levels in p53-null, wild-type, and p53^Δ*31*/Δ*31*^ MEFs that expressed C-terminally truncated p53 protein with increased p53 activity (Simeonova et al. 2013). Since p53-mediated down-regulation of most genes is indirect, requiring the Cdk inhibitor p21 (Löhr et al. 2003), we also included p21-null MEFs in our analysis. Across the p53 allelic series of MEFs, mRNA levels corresponding to *Cenpa* and *Hjurp* varied inversely with basal p53 activity (Fig. 2A). This type of inverse correlation in mRNA expression compares with previously characterized p53-repressed genes such as *Fancd2* (Jaber et al. 2016). Furthermore, the treatment of cells with nutlin, a drug that activates p53 by preventing its interaction with the ubiquitin ligase Mdm2, further showed that down-regulation of the two genes ensues upon p53 activation (Fig. 2A). In agreement with an indirect p53-dependent effect, the response in *Cenpa* and *Hjurp* expression was attenuated, if not completely abrogated, in p21-null cells (Fig. 2A). This p53-mediated regulation is also conserved in human cells, as nutlin led to the down-regulation of *CENPA* and *HJURP* in the MRC5 normal human fibroblasts but not in their SV40 transformed derivative cells, SVM, in which p53 is inactive (Fig. 2B).

Indirect p53-mediated gene repression of cell cycle genes whose promoters contain CDE/CHR regulatory motifs occurs through the recruitment of the DREAM repressor complex at their promoters (Benson et al. 2014). Interestingly, a recent meta-analysis that combined chromatin immunoprecipitation (ChIP) data with in silico analyses suggested that the human genes *CENPA* and *HJURP* are possible DREAM-regulated genes (Fischer et al. 2016). We thus hypothesized that down-regulation of HJURP and CENP-A by p53 could depend on the presence of CDE/CHR motifs. We thus used a positional frequency matrix designed to search for CDE/CHR motifs in silico (Jaber et al. 2016) and identified potential CDE/CHR at the promoters of *Cenpa* and *Hjurp*, close to each transcription start site (TSS) (Fig. 2C; Supplemental Fig. 2A). We found that mutating the putative CHR element affected the nutlin-dependent repression of the *Cenpa* promoter dramatically but not completely (Fig. 2D). However, the nutlin-dependent repression of the *Hjurp* promoter was completely abrogated by mutating the putative CDE element (Fig. 2E). These changes, observed in cell populations that harbor similar cell cycle distribution (Supplemental Fig. 2C), provide evidence for the functional role of the CDE/CHR motifs that we identified in silico. Thus, we conclude that the presence of responsive CDE/CHR motifs provides a relevant molecular target with critical roles in the down-regulation of *Cenpa* and *Hjurp* upon p53 activation.

### CENP-A and HJURP are not typical drivers of cellular transformation

**Figure 3.**
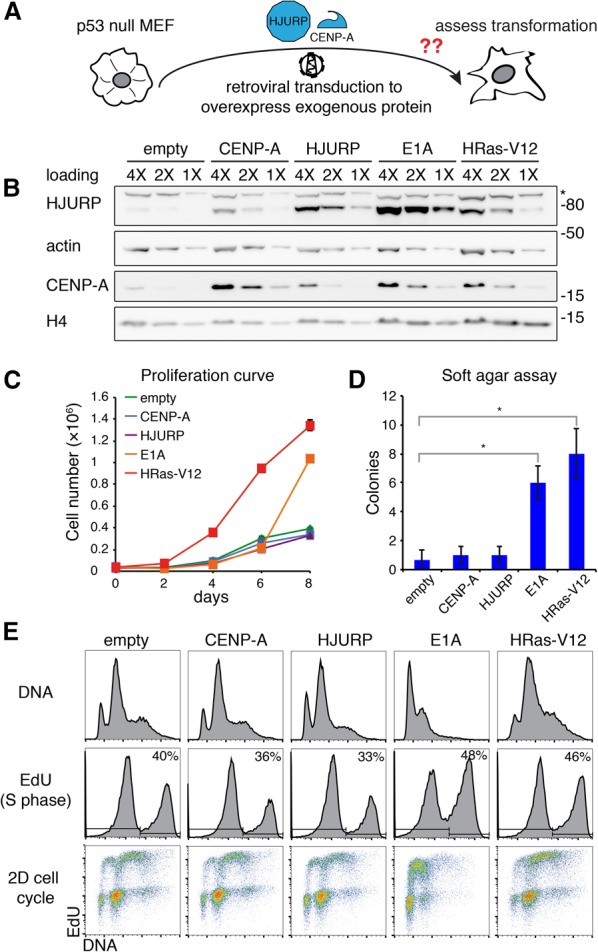
CENP-A or HJURP overexpression does not alter proliferation or cell cycle in p53-null MEFs. (*A*) Scheme outlining an experimental approach. Immortalized p53-null MEFs were transduced with retroviral particles encoding HJURP or CENP-A, expressed under the control of the retroviral promoter, and assessed for transformation hallmarks such as increased cell proliferation or substrate-independent cell growth. (*B*) Western blot of HJURP and CENP-A levels in RIPA-soluble extracts from p53-null MEFs transduced with the indicated retroviral construct 6 d after transduction and following hygromycin selection. H4 levels are also shown. Actin was used as a loading control. An asterisk marks a nonspecific band detected with the HJURP antibody. A twofold dilution series of each extract is represented by 4X, 2X, and 1X. Molecular weight protein markers are indicated at the *right*. (*C*) Proliferation curve of p53-null MEFs transduced with the indicated retroviral construct. MEFs (3 × 10^4^) were seeded in 1 mL of growth medium on 24-well plates. The graph displays the quantified cell number ± SEM of triplicates. (*D*) Soft agar colony-forming assay in p53-null MEFs transduced with the indicated retroviral construct. We stained colonies with Sytox Green 4 wk after seeding. Bars represent quantification of visible colony numbers ± SEM. *n* = 3. (*) *P* < 0.05, significance determined by a *t*-test. (*E*, *top* panels) Cell cycle analysis by flow cytometry in p53-null MEFs transduced with the indicated retroviral construct following EdU uptake and PI (DNA) staining. The percentage of EdU-positive cells (S phase) is indicated.

To test whether CENP-A or HJURP up-regulation can bypass oncogenic signaling and directly drive cellular transformation on its own, we overexpressed these factors by retroviral transduction of p53-null MEFs. We then assessed these cells for transformation hallmarks using E1A or HRas-V12 transduced cells as controls (Fig. 3A). Interestingly, overexpression of exogenous CENP-A resulted in an increase of endogenous HJURP (Fig. 3B). Likewise, overexpression of exogenous HJURP resulted in increased levels of endogenous CENP-A, suggesting that each protein reciprocally stabilizes its binding partner and a possible coregulation mechanism. As controls for effective drivers, we verified that E1A and HRas-V12 transduced p53-null MEFs exhibited increased proliferation relative to empty vector (Fig. 3C) and were able to form colonies in soft agar (Fig. 3D; Supplemental Fig. S3A). However, independent overexpression of CENP-A or HJURP in p53-null MEFs did not alter proliferation rates compared with cells transduced with empty vector, and cells were not capable of anchorage-independent growth (Fig. 3C,D; Supplemental Fig. S3A). Additionally, they showed no increase in the proportion of S-phase cells within the first two passages after transduction (Fig. 3E). These results show that overexpressing these two centromere proteins along with loss of p53 function is not sufficient for transformation to occur.

We also compared the localization of exogenously overexpressed CENP-A with endogenous CENP-A, which is up-regulated as a consequence of either HJURP overexpression or oncogenic transformation. We detected exogenous CENP-A at centromeres on minor satellite DNA repeats but also on chromosome arms (Supplemental Fig. S3B). This resembles the ectopic incorporation throughout euchromatin previously described upon CENP-A overexpression in HeLa cells (Lacoste et al. 2014). Interestingly, HJURP-overexpressing cells showed an increased intensity of CENP-A signal at centromeric regions but no ectopic localization (Supplemental Fig. S3B). The coup-regulation observed as a result of E1A or HRas-V12 transformation led to a similar localization exclusive to centromeres with an increased intensity (Supplemental Fig. S3B). This suggests that the dosage of the CENP-A variant relative to its dedicated chaperone is important for the ultimate localization of up-regulated CENP-A and underscores the specificity of HJURP to target CENP-A to centromeres. Furthermore, the capacity to reinforce CENP-A deposition could lead to enhanced centromere function by providing additional anchoring points for kinetochore assembly.

### HJURP is required for the survival of hyperproliferating cells: HJURP knockout strategy using CRISPR/Cas9

Given the prevalence of HJURP (and CENP-A) overexpression upon loss of p53 and oncogenic transformation, we hypothesized that transformed cells may develop a dependency on increased HJURP levels. To test this hypothesis, we sought to compare the effects of HJURP depletion in cells with functional p53 with cells that have lost p53 and/or undergone oncogenic transformation. However, multiple duplication events encompassing the *Hjurp* locus in mice (Keane et al. 2011; Pezer et al. 2015) challenged traditional depletion strategies. We characterized the *Hjurp* copy number in higher resolution by analyzing next-generation sequencing data in four mouse strains: two classical laboratory strains (C57BL/6 and BALB/cJ) and two wild-derived strains (CAST/EiJ and SPRET/EiJ) (Supplemental Fig. S4A). An approximately fivefold to sixfold increase in copy number occurs over a 100-kb region including *Hjurp* in C57BL/6, BALB/cJ, and CAST/EiJ mice but not in SPRET/EiJ mice (Supplemental Fig. S4A). DNA FISH revealed the unmapped *Hjurp* paralogs adjacent to the centromeric repeats of the chromosome 1 (which also contains the mapped *Hjurp* gene) and to the centromere of a distinct chromosome pair (Supplemental Fig. S4B). The high degree of conservation among the paralogs (data not shown) suggested that a single-guide RNA (sgRNA) could potentially knock out multiple *Hjurp* copies using CRISPR–Cas9 technology.

**Figure 4.**
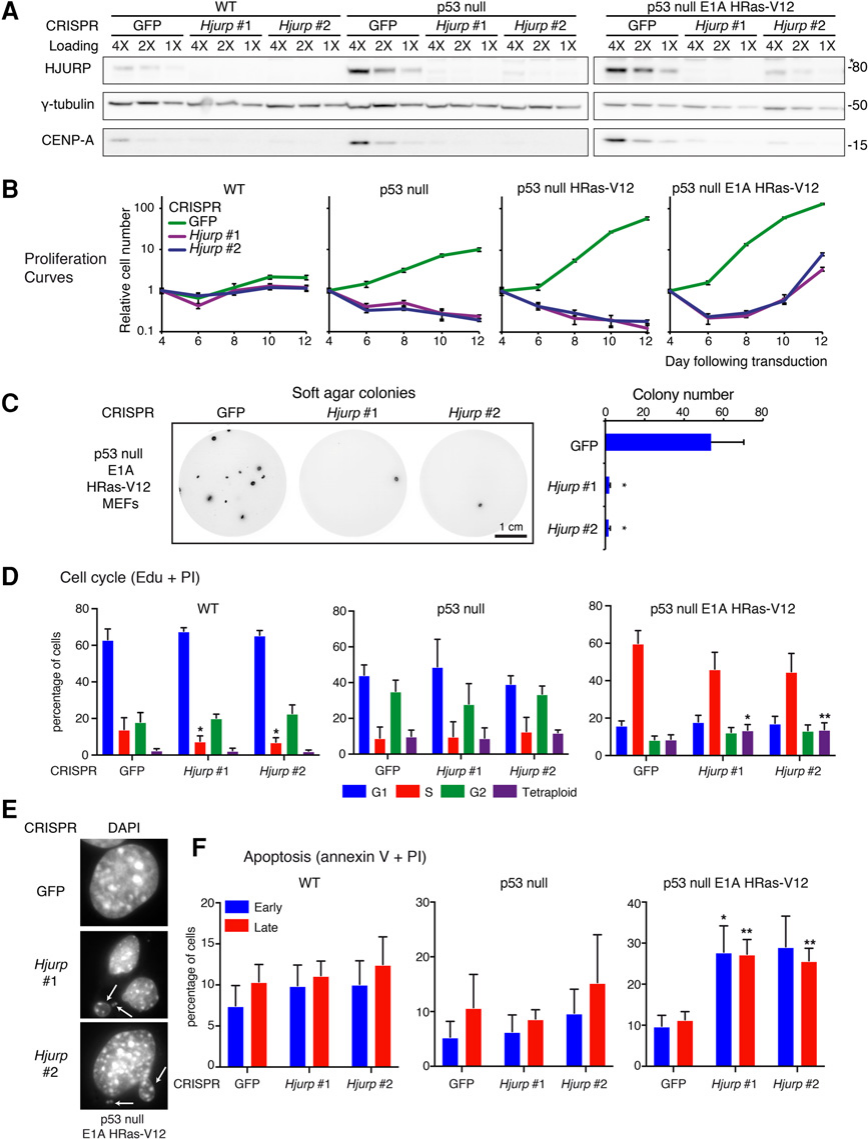
Transformed cells require HJURP for growth and survival. (*A*) Western blot of HJURP and CENP-A levels in wild-type, p53-null, or p53-null E1A and HRas-V12 transformed MEFs 6 d after transduction with CRISPR lentiviral particles against GFP (control) or two sgRNA constructs targeting *Hjurp* (*Hjurp* #1 and *Hjurp* #2) following puromycin selection. γ-Tubulin is used as a loading control. An asterisk marks a nonspecific band detected with the HJURP antibody. A twofold dilution series of each extract is represented by 4X, 2X, and 1X. Molecular weight protein markers are indicated at the *right*. (*B*) Proliferation assays in MEFs of the indicated genotypes following CRISPR-mediated depletion of *Hjurp*. MEFs (3 × 10^4^) were seeded in triplicate in 1 mL of growth medium on 24-well plates 4 d after lentiviral transduction of CRISPR constructs. *Hjurp* CRISPR-resistant clones begin to emerge in E1A/HRas-V12 transformed p53-null MEFs 12 d after transduction. Results represent mean cell number ± SEM of triplicates relative to day 1 of the experiment and are shown in log scale. (*C*) Soft agar assay of p53-null E1A HRas-V12 transformed MEFs transduced with control (GFP) or *Hjurp* CRISPR constructs. We stained colonies with Sytox Green 4 wk after cell seeding. Bars represent quantification of visible colony numbers ± SEM. *n* = 3. *P* < 0.05, *t*-test. (*D*) Quantification of cell cycle analysis by flow cytometry (Edu/PI staining) in wild-type, p53-null, or p53-null E1A HRas-V12 transformed MEFs transduced with CRISPR constructs. Mean percentages of cells ± SEM for G1, S, G2/M, and tetraploid/aneuploid (>4N) populations (*n* = 3) are shown. Statistical significance is shown where relevant. (*) *P* < 0.05; (**) *P* < 0.005, *t*-test. See also Supplemental Figure S4D for corresponding flow cytometry plots. (*E*) Immunofluorescence images of p53-null E1A HRas-V12 transformed MEFs at day 6 after transduction with CRISPR constructs following puromycin selection. We stained nuclei with DAPI. See also Supplemental Figure S4F for full images. Individual magnified nuclei are shown. Micronuclei are highlighted by arrows. (*F*) Quantification of apoptosis by flow cytometry (Annexin V/PI staining) in wild-type, p53-null, or p53-null E1A HRas-V12 transformed MEFs transduced with CRISPR constructs. Mean percentages of cells ± SEM in early or late apoptosis are shown. Statistical significance is shown where relevant. (*) *P* < 0.05; (**) *P* < 0.005, *t*-test. See also Supplemental Figure S4E for corresponding flow cytometry plots.

We designed two sgRNA sequences targeting conserved sequences in exons 1 and 2 of the *Hjurp* paralogs (Supplemental Fig. S4C). We transduced wild-type, p53-null, or p53-null HRas-V12 or E1A/HRas-V12 transformed MEFs with lentiviral particles encoding CRISPRs against GFP (as a nontargeting control) or *Hjurp* (*Hjurp* #1 and *Hjurp* #2) and achieved an almost complete depletion of HJURP (Fig. 4A). Upon HJURP loss, we observed a corresponding depletion in endogenous CENP-A levels, confirming that CENP-A deposition and stability depend on the presence of HJURP (Fig. 4A).

As expected, HJURP depletion led to a reduction in the proliferative capacity in all MEF cells tested due to the essential role of CENP-A in mitotic progression, consistent with previous studies (Dunleavy et al. 2009; Foltz et al. 2009; Fachinetti et al. 2013). However, we observed that the decrease in proliferation remained limited in wild-type MEFs (Fig. 4B), in part since they proliferate slowly and also because they are prone to enter replicative senescence or cell cycle arrest in response to p53 activation. Importantly, in p53-null MEFs, HJURP-depleted cells not only stopped proliferating but also underwent cell death (Fig. 4B). This result also held true in p53-null MEFs further transduced with HRas-V12 or a combination of E1A and HRas-V12. To assess the impact of HJURP loss on the ability of transformed cells to sustain growth in the absence of substrate attachment, we carried out a soft agar assay. While the p53-null E1A HRas-V12 transformed MEFs transduced with the control GFP targeting CRISPR construct formed numerous colonies, colony number dropped significantly upon HJURP depletion (Fig. 4C).

HJURP depletion also particularly impacted the cell cycle in p53-null transformed cells compared with wild-type or p53-null MEFs. In wild-type MEFs, HJURP depletion resulted in a slight increase in the G1 population and a statistically significant decrease in S phase. These mild changes could be due to the slow proliferation rate of these cells in general. Interestingly, alterations in cell cycle following HJURP depletion in p53-null MEFs were minimal (Fig. 4D; Supplemental Fig. S4D). This suggests that a potential p53-dependent arrest response elicited by abnormal HJURP/CENP-A levels cannot take place in p53-null cells. Strikingly, in p53-null E1A HRas-V12 transformed MEFs, which overexpress HJURP and CENP-A to the highest levels, HJURP depletion resulted in an increase of cells in G1 and G2/M and reduced S phase, highlighting impaired proliferation. While p53-null MEFs display basal aneuploidy, this aneuploidy significantly increased following HJURP depletion, manifesting as both an increase in polyploidy and a broadened DNA content distribution, evident in G1 (Fig. 4D; Supplemental Fig. S4D). To further characterize the potential effect of aneuploidy in p53-null E1A HRas-V12 transformed MEFs, we examined these cells by immunofluorescence. Upon HJURP depletion in these cells, we observed a range of nuclear abnormalities, including increased frequency of micronuclei, consistent with aneuploidy (Fig. 4E; Supplemental Fig. S4F,G). Additionally, we observed that a proportion of cells depleted for HJURP had severely enlarged multiple nuclei with a tightly associated α-tubulin network at the nuclear periphery, suggesting that defects in chromosome segregation had occurred (Supplemental Fig. S4F,G).

To further characterize how cell death contributed to loss of proliferative capacity in the different MEF genotypes depleted for HJURP, we examined whether these cells underwent apoptosis using Annexin V labeling and flow cytometry. In wild-type and p53-null MEFs, no significant increase in apoptosis was observed upon HJURP depletion. This suggests that apoptosis-independent cell death could contribute to loss of proliferative capacity, particularly in the p53-null MEFs. In p53-null E1A/HRas-V12 transformed cells, however, we detected a significant increase in both early and late apoptosis relative to the GFP CRISPR control (Fig. 4F; Supplemental Fig. S4E). Collectively, these results suggest that p53-null transformed cells are particularly sensitive to HJURP depletion, which, in this context, leads to severe perturbations in proliferative capacity and chromosome number and, ultimately, p53-independent apoptosis. Altogether, these results strongly support the hypothesis that transformed cells become addicted to high levels of HJURP.

### p53 is activated in response to HJURP depletion and induces cell cycle arrest

**Figure 5.**
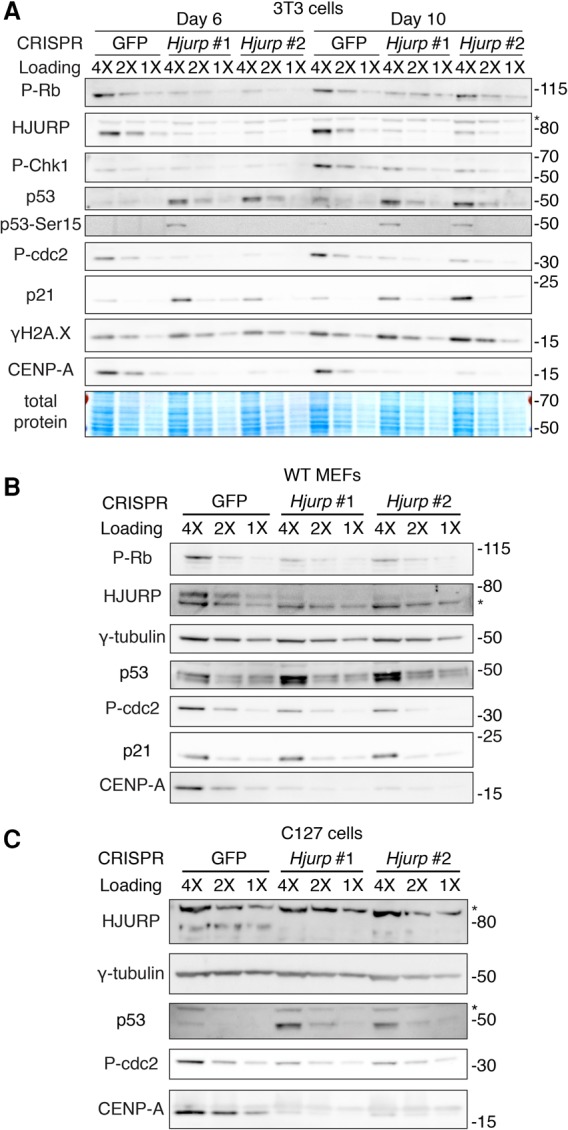
In cells with functional p53, p53 is up-regulated upon *Hjurp* knockout, and cells undergo cell cycle arrest. (*A*) Western blot in RIPA-soluble extracts of NIH/3T3 cells 6 or 10 d after CRISPR lentiviral transduction and following puromycin selection. We analyzed HJURP, CENP-A, p53, phospho-p53, and p21 levels and markers of cell cycle arrest (p-Rb and p-cdc2) or DNA damage (P-Chk1 and γH2A.X). An asterisk marks a nonspecific band detected with the HJURP antibody. A twofold dilution series of each extract is represented by 4X, 2X, and 1X. Total protein was detected with Memcode protein stain. Molecular weight protein markers are indicated at the *right*. (*B*) Western blot in RIPA-soluble extracts of wild-type MEFs 6 d after CRISPR lentiviral transduction and following puromycin selection. An asterisk marks a nonspecific band detected with the HJURP antibody. γ-Tubulin was used as a loading control. A twofold dilution series of each extract is represented by 4X, 2X, and 1X. Molecular weight protein markers are indicated at the *right*. (*C*) Western blot in RIPA-soluble extracts of C127 mouse mammary epithelial immortalized cells 6 d after CRISPR lentiviral transduction and following puromycin selection. An asterisk marks a nonspecific band detected with the HJURP antibody. γ-Tubulin was used as a loading control. A twofold dilution series of each extract is represented by 4X, 2X, and 1X. Molecular weight protein markers are indicated at the *right*.

To determine whether, in addition to repressing HJURP and CENP-A at the transcriptional level, the p53 pathway could be activated upon loss of HJURP and CENP-A, we also examined cell lines that express p53 but, unlike wild-type MEFs, are not prone to enter replicative senescence. Again, using CRISPR/Cas9 technology, we ablated *Hjurp* in NIH/3T3 mouse fibroblasts, which are highly proliferative but have not lost p53 expression. At day 6 and day 10 following CRISPR transduction, we observed an increase in p53 levels (Fig. 5A). We also observed an increase in p53 phosphorylation (Ser15) and p21 levels, confirming stabilization of the p53 protein and activation of the p53 growth arrest pathway. To test whether p53 up-regulation was related to a DNA damage response to CRISPR activity targeting the multiple paralogs of *Hjurp*, we measured levels of Chk1 phosphorylation and γH2A.X and observed no differences upon HJURP depletion (Fig. 5A). However, we did detect a decrease in Rb and Cdc2 phosphorylation, suggesting that p53 mediates cell cycle arrest upon HJURP depletion (Fig. 5A). We confirmed these results with a rescue experiment in which we transduced NIH/3T3 cells with a doxycycline-inducible CRISPR-resistant *Hjurp* transgene followed by transducing the cells with CRISPR constructs against endogenous *Hjurp* (scheme in Supplemental Fig. S5A). We did not detect p53 accumulation in the presence of doxycycline, but, 6 d following doxycycline withdrawal (HJURP off), p53 levels increased with *Hjurp* CRISPRs compared with GFP (Supplemental Fig. S5B). We also detected accumulation of p53 upon HJURP depletion in wild-type MEFs and a decrease in Rb and Cdc2 phosphorylation, reflecting cell cycle arrest (Fig. 5B). In the nontransformed mouse mammary cell line C127, we detected p53 accumulation and a concomitant reduction of phosphorylated Cdc2 upon HJURP depletion, again confirming cell cycle arrest (Fig. 5C). Indeed, cell cycle analysis revealed that cells depleted for HJURP do not display aneuploidy but arrest in G1 and G2/M phase (Supplemental Fig. S5C).

### p53 prevents the accumulation of aneuploidy induced by the loss of HJURP

**Figure 6.**
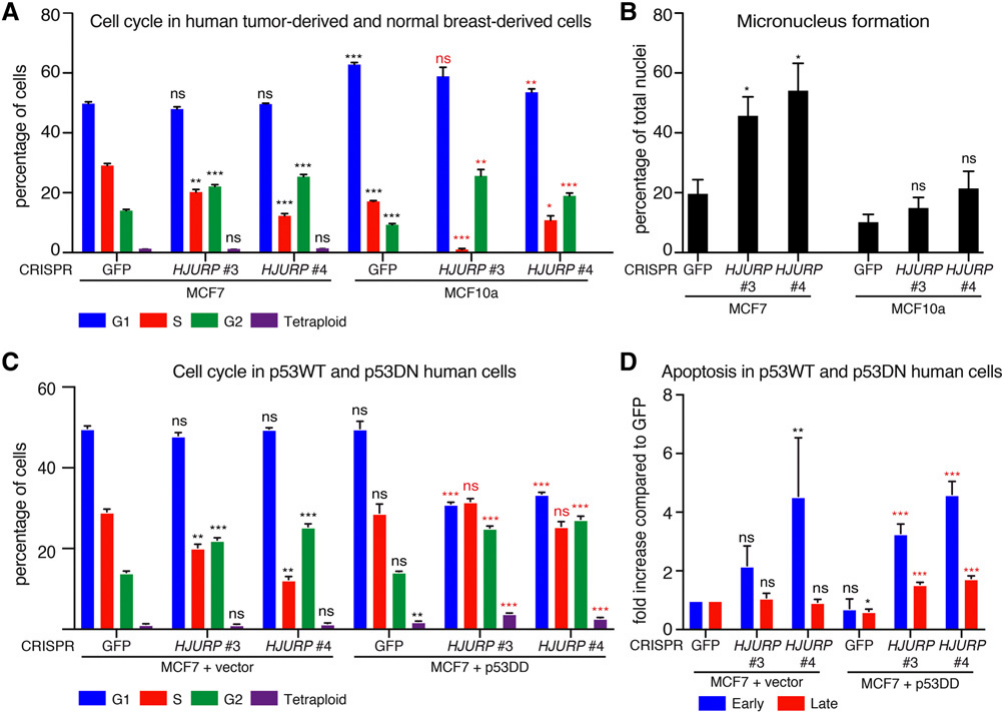
Human transformed MCF7 cells display aneuploidy following HJURP depletion. (*A*) Quantification of cell cycle analysis by flow cytometry (Edu/PI) in MCF7 human breast tumor-derived cells or MCF10a immortalized nontransformed breast cells, both with intact p53, 6 d after CRISPR lentiviral transduction and following puromycin selection. Mean percentages of cells ± SEM of triplicates are shown for G1, S, G2/M, and tetraploid/aneuploid (>4N) populations. Statistical significance is shown where relevant. (*) *P* < 0.05; (**) *P* < 0.005; (***) *P* < 0.0005, *t*-test. (Black asterisks) Significance compared with GFP control in MCF7 cells; (red asterisks) significance compared with GFP control in MCF10a cells. (*B*) Quantification of micronucleus formation in MCF7 cells 6 d after CRISPR lentiviral transduction and following puromycin selection. Nuclei were stained with DAPI; at least 200 nuclei per CRISPR condition were counted. See Supplemental Figure S6B for corresponding immunofluorescence images. (*C*) Quantification of cell cycle analysis by flow cytometry (Edu/PI) in MCF7 cells transduced with a control vector (MCF7 + vector) or p53 DD 6 d after CRISPR lentiviral transduction and following puromycin selection. Mean percentages of cells ± SEM of triplicates are shown for G1, S, G2/M, and tetraploid/aneuploid (>4N) populations. Statistical significance is shown where relevant. (**) *P* < 0.005; (***) *P* < 0.0005, *t*-test. (Black asterisks) Significance compared with GFP control in MCF7 control cells; (red asterisks) significance compared with GFP control in MCF7 + p53 DD cells. (*D*) Quantification of apoptosis by flow cytometry (Annexin V/PI) in MCF7 cells transduced with a control vector (MCF7 + vector) or p53 DD 6 d after CRISPR lentiviral transduction and following puromycin selection. Mean percentages of cells ± SEM of triplicates are shown for G1, S, G2/M, and tetraploid/aneuploid (>4N) populations. Statistical significance is shown where relevant. (*) *P* < 0.05; (***) *P* < 0.0005, *t*-test. (Black asterisks) Significance compared with GFP control in MCF7 control cells; (red asterisks) significance compared with GFP control in MCF7 + p53 DD cells.

We wished to determine whether dependency on HJURP was conserved in human cells and whether the transformation state of cells played a role in increasing their sensitivity to HJURP depletion. First, we compared the effect of HJURP depletion in tumor-derived MCF7 cells with MCF10a cells, an immortalized breast cell line derived from nontumor tissue, both of which have intact p53. We selected two CRISPR sequences targeting human HJURP (*HJURP* #3 and *HJURP* #4), which effectively depleted HJURP (Supplemental Fig. S6A). HJURP depletion in both MCF7 cells and MCF10a cells led to loss of proliferative capacity, highlighted by reduced S phase and accumulation in G2/M (Fig. 6A). We examined both cell lines by immunofluorescence following HJURP depletion and observed a striking enlargement of nucleus and micronucleus formation following HJURP depletion exclusively in the tumor-derived MCF7 cell line and not in the MCF10a cell line, suggesting an accumulation of aneuploidy (Fig. 6B; Supplemental Fig. S6B). We also depleted HJURP in MCF7 cells expressing vector alone (MCF7 + vector) or a p53 dominant-negative mutant that mimics p53 loss (p53 DD) (Hahn et al. 2002) and examined changes in cell cycle. The expression of the p53 DD mutant completely abrogates wild-type p53 activity, as shown by loss of p21 activation upon nutlin treatment in these cells compared with the MCF7 + vector control cells (Supplemental Fig. S6C). Consistent with loss of p53 activity, we observed increased HJURP and CENP-A levels in MCF7 + p53 DD cells transduced with control GFP CRISPR compared with MCF7 + vector cells transduced with control GFP CRISPR. We demonstrated efficient HJURP depletion and a corresponding decrease in CENP-A levels in both control and p53-DD-expressing MCF7 cell lines transduced with HJURP CRISPRs (Supplemental Fig. S6D). MCF7 cells expressing p53 DD did display G2/M accumulation following HJURP depletion; however, S phase was unaffected, allowing accumulation of polyploidy (Fig. 6C). MCF7 + p53 DD cells displayed significantly higher apoptosis following HJURP depletion than MCF7 + vector cells (Fig. 6D). Collectively, these results suggest that, in p53-proficient cells, p53 can maintain genome integrity when HJURP is depleted by inducing cell cycle arrest. However, p53-null transformed cells experience severe aneuploidy upon loss of HJURP function and thus are particularly sensitive to HJURP depletion.

### In vivo HJURP depletion in established tumors results in their regression

**Figure 7.**
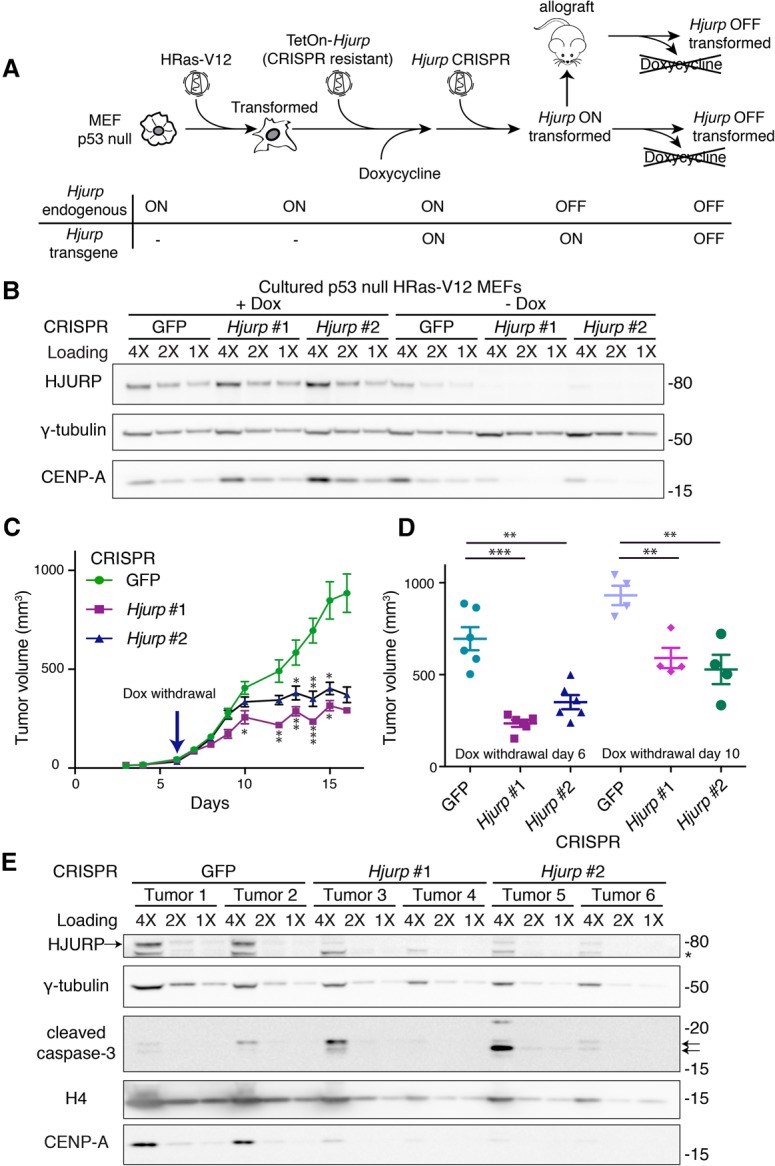
Inducible HJURP loss in an established tumor leads to growth arrest. (*A*) Scheme depicting the approach to generate transformed MEFs with inducible *Hjurp* rescue for allograft assays. Cultured p53-null MEFs are transduced first with retroviruses encoding HRas-V12, which results in cellular transformation, and then with lentiviruses encoding a CRISPR-resistant *Hjurp* transgene with the TetON promoter. Endogenous *Hjurp* paralogs were then inactivated using CRISPR constructs in the presence of doxycycline (Dox), allowing the MEFs to survive and expand. These cells, grown in the presence of doxycycline (*Hjurp* on), can be injected into mice and form tumors. Doxycycline withdrawal reverts the rescue and allows temporal control of *Hjurp* knockout in either cell culture or allograft assays. (*B*) Western blot of HJURP and CENP-A levels in RIPA-soluble extracts of cultured MEFs generated as described above in *A* and grown in either the presence of doxycycline or after 8 d of doxycycline withdrawal. γ-Tubulin was used as a loading control. A twofold dilution series of each extract is represented by 4X, 2X, and 1X. Molecular weight protein markers are indicated at the *right*. (*C*) Allograft assay measuring tumor growth following HJURP knockout over time. We generated p53-null HRas-V12 MEFs with an inducible CRISPR-resistant HJURP transgene maintained in the presence of doxycycline, as described in *A*. We subcutaneously injected 1 million cells into each flank of nude mice (three mice per CRISPR construct). We withdrew doxycycline on day 6 after injection (*Hjurp* switched off) and measured allograft tumor volume over time. Data represent mean tumor volume ± SEM. (*) *P* < 0.05; (**) *P* < 0.005; (***) *P* < 0.0005, *t*-test. (*D*) Comparison of different time courses of doxycycline withdrawal (*Hjurp* switched off) from two allograft experiments. We withdrew doxycycline on either day 6 or day 10 after injection. Allograft tumor volume on day 14 or 19, respectively, is shown (the latest time point in the experiment where all control mice remained viable). Data represent mean tumor volume ± SEM at the conclusion of the experiment. (**) *P* < 0.005; (***) *P* < 0.0005, *t*-test. (*E*) Western blot on RIPA-soluble protein extracts from the subcutaneous tumors excised at the end of the allograft assay. Tissues from two different tumors were analyzed for each CRISPR construct. We assessed HURP and CENP-A levels as well as histone H4 and cleaved caspase-3 (highlighted with arrows). An asterisk marks a nonspecific band detected by the HJURP antibody. The HJURP band is indicated by an arrow at the *left*. γ-Tubulin was used as a loading control. A twofold dilution series of each extract is represented by 4X, 2X, and 1X. Molecular weight protein markers are indicated at the *right*.

We hypothesized that if p53-deficient tumor cells become dependent on HJURP in order to maintain a state of hyperproliferation, then HJURP depletion in an established p53-null tumor could block its progression. To test this hypothesis, we established a system allowing us to induce HJURP depletion in a tumor of measurable size. We transduced p53-null HRas-V12 transformed MEFs with a doxycycline-inducible CRISPR-resistant HJURP transgene. We induced transgene expression and then knocked out endogenous *Hjurp* paralogs using CRISPR constructs (experimental outline in Fig. 7A). Six days following doxycycline withdrawal, the transgene expression was switched off, and endogenous HJURP reached nondetectable levels in MEFs in culture (Fig. 7B). Eight days following doxycycline withdrawal, we observed a reduction in the proportion of cells in S phase and a strong accumulation of aneuploidy (broadened DNA content distribution) and apoptosis in HJURP knockout cells compared with cells targeted with the GFP control (Supplemental Fig. S7A,B).

Using this system, we conducted two separate allograft assays in nude mice, where we injected these cells in the presence of doxycycline (HJURP on), allowed tumors to develop, and then withdrew doxycycline either 6 or 10 d after injection of the cells (HJURP off). We monitored tumor growth over time and observed that, in the mice injected with HJURP CRISPR cells, the tumor size reached a plateau at around day 10 following injection (doxycycline withdrawal day 6) (Fig. 7C), while the tumors targeted with the GFP control continued to grow and compromised animal survival. The difference in tumor size at the end of both experiments was statistically significant for both *Hjurp* CRISPR constructs compared with the GFP control (Fig. 7D). Earlier withdrawal of doxycycline (day 6) resulted in faster tumor growth arrest, and mouse health remained unaffected by the tumor burden compared with the GFP control mice, which lost weight (data not shown). We confirmed HJURP depletion in these tumors by Western blot, and CENP-A levels were also significantly reduced (Fig. 7E). Interestingly, histone H4 levels were also slightly reduced following HJURP depletion, consistent with the contribution of histone H4 to CENP-A-containing nucleosomes. We examined CENP-A levels by immunofluorescence in tissue sections obtained from tumors. We observed a decrease in the number and intensity of CENP-A foci detected in the HJURP off tumors compared with GFP control tumors (Supplemental Fig. S7C,D). Quantification of the number of foci in the nuclei of tumor cells revealed a twofold decrease in HJURP off tumors (Supplemental Fig. S7D). Interestingly, GFP control tumors had about twice the number of CENP-A foci as adjacent tissue, and HJURP depletion reduced focus number to levels similar to that of adjacent normal tissue (Supplemental Fig. S7E).

We also examined markers of apoptosis, proliferation, and DNA damage in tumor samples by immunofluorescence. Large tumor areas were characterized by cleaved caspase-3 staining, indicating ongoing cell death (Supplemental Fig. S7F). Consistent with the observed block in tumor growth, *Hjurp* depletion led to a marked decrease in proliferation, as determined by BrdU incorporation assay (Supplemental Fig. S7G,H). However, examination of tumor lysates by immunoblotting detected a variable degree of apoptosis in individual tumors (Fig. 7E), probably reflecting the nonhomogenous distribution of dead cells. Closer inspection of viable areas from tumor sections revealed that more cells were positive for cleaved caspase-3 and γH2A.X in the *Hjurp*-depleted tumors than in the control tumors (Supplemental Fig. S7G,I,J), suggesting that loss of HJURP may also lead to genome instability and apoptosis. Collectively, these results in vivo support the view that transformed cells become dependent on increased CENP-A and HJURP levels to maintain a hyperproliferative state.

## Discussion

Centromeric factors are emerging players in cancer biology as both prognostic markers and potential therapeutic targets (Liu and Yen 2009; Tomonag 2009; Carone and Lawrence 2013; Filipescu et al. 2014; Mahadevan et al. 2014). Among these, CENP-A and HJURP levels are elevated in human cancers, and both factors have been put forward as prognostic and predictive biomarkers (Valente et al. 2013; Montes de Oca et al. 2015; Sun et al. 2016). The role of p53 in chromatin regulation has been emerging recently. Gain-of-function p53 mutations can up-regulate key chromatin regulators such as MLL1 and MLL2, changing the histone methylation and acetylation landscape in cells (Zhu et al. 2015). Furthermore, p53 can trigger cell cycle arrest in response to nucleosome depletion (Sokolova et al. 2017). In cancer cells lacking p53, this results in prolonged S phase and eventual cell death. p53 is thus an important sensor of altered chromatin landscape, and loss-of-function or gain-of-function mutations in p53 frequently lead to chromatin alterations that impact tumor evolution. Given the established link between a p53-mediated senescence response in normal primary human cells upon imbalance of HJURP or CENP-A levels (Maehara et al. 2010; Heo et al. 2013), we explored whether CENP-A and HJURP gene expression was specifically up-regulated in human cancers lacking functional p53 (Fig. 1). Our initial analysis added to the evidence that this tumor suppressor could play a role in the regulation of HJURP and CENP-A.

We initially sought to understand what might cause the up-regulation of CENP-A and HJURP, frequently observed in human cancers. While copy number variations of *HJURP* and *CENPA* can occur in tumors and could possibly explain certain cases of overexpression, the *HJURP* locus in humans has not undergone duplication events as in mice (L Wilson, unpubl.). We show here that p53 loss correlates with increased CENP-A and HJURP mRNA levels in both MEFs (Fig. 3A,B) and human cancers (Fig. 1A; Supplemental Fig. S1). These data suggested that *HJURP* and *CENPA* genes could be repressed by intact p53 in normally proliferating cells. Interestingly, we also observed increased *HJURP* and *CENPA* mRNA levels in pooled cancers with p53 gain-of-function mutations (R175H, R248Q, R248W, R249S, and R273H) (Supplemental Fig. 1). It was thus important to determine how p53-dependent regulation of *HJURP* and *CENPA* was occurring. As is often the case for p53-repressed genes (Beckerman and Prives 2010; Quaas et al. 2012), p53-mediated gene suppression occurs indirectly via p21 and often involves the recruitment of the E2F4-containing complex DREAM at CDE/CHR motifs (Fig. 2). A subset of DREAM targets is also bound by FOXM1, controlling expression of genes in G2/M (Fischer et al. 2016). The identification of putative CDE/CHR motifs within the promoters of CENP-A and HJURP (Fig. 2) enabled us to assess their functionality by mutagenesis. Remarkably, on the one hand, mutation of the CDE element at the *Hjurp* promoter completely abolished its repression in response to nutlin (Fig. 2E), suggesting that *Hjurp* regulation occurs predominantly via binding of the E2F4–DREAM complex. On the other hand, mutating the CHR element of *Cenpa* significantly but incompletely affected the nutlin-dependent repression of this promoter (Fig. 2D), suggesting that another level of promoter regulation may exist, perhaps via binding to the adjacent CDE motif, which may be functional despite its imperfect match to the consensus. Considering that FOXM1 binds upstream of both the HJURP and CENP-A promoters and is itself repressed by p53 (Wang et al. 2005, 2013; Grant et al. 2013; Zhang et al. 2014; Yau et al. 2015), it is tempting to speculate that FOXM1 could also contribute to a positive feedback loop of gene activation upon p53 loss. Interestingly, loss of FOXM1 is associated with phenotypes reminiscent of CENP-A loss, such as mitotic catastrophe (Wonsey and Follettie 2005). Certain tumor growth-promoting genes and cancer down-regulated microRNAs have also been proposed recently to regulate CENP-A expression (Sun et al. 2016). Identifying how these factors cooperate will be necessary to define the best combinatorial targeting approaches with the aim to prevent abnormal accumulation of centromeric factors in cancer cells.

Once up-regulation at the RNA level has occurred following p53 loss, a second level of control exists whereby HJURP and CENP-A reciprocally stabilize the other partner of the histone/chaperone complex. We consistently observed that overexpression or depletion of one partner leads to the reciprocal increase or decrease, respectively, of the other. We also show that siRNA-mediated depletion of CENP-A led to proteasome-dependent degradation of HJURP (Supplemental Fig. S1E). This protection mechanism at the protein level could contribute to the relatively long turnover period of CENP-A. Thus, in addition to HJURP and CENP-A regulation at their promoters, once one partner is up-regulated at the protein level, it can in turn stabilize the other and further protect from proteasomal degradation.

**Figure 8.**
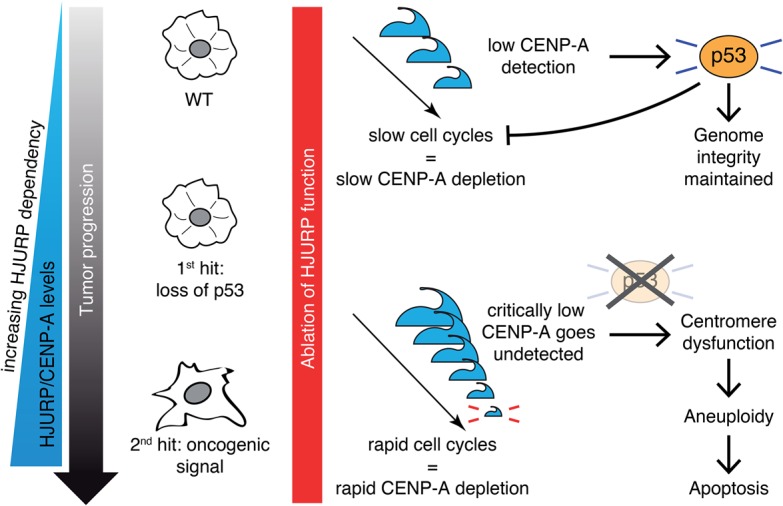
Proposed model: HJURP sustains cellular transformation in cells lacking p53. HJURP and CENP-A levels are increased in cells that lose p53 expression and increase even further following oncogenic transformation. When HJURP is depleted in wild-type cells with functional p53, p53 senses gradual CENP-A depletion and induces cell cycle arrest in order to maintain genome integrity. In cells lacking p53, HJURP depletion results in rapid CENP-A loss at centrosomes, leading to centrosome dysfunction, aneuploidy, and p53-independant apoptosis.

Cancer cells also have to reconcile their rapid cycling speed with the short time window, normally limited to early G1, when HJURP mediates CENP-A deposition (Jansen et al. 2007; Silva et al. 2012; Müller et al. 2014). Thus, cancer cells would adapt to more frequent rounds of replication and mitosis by up-regulating CENP-A and HJURP protein levels to increase the efficiency of centromere propagation, similar to a mechanism of nononcogene addiction (Luo et al. 2009). While we showed that wild-type MEFs and highly proliferating cell lines that have preserved p53 function stopped proliferating but did not die significantly upon *Hjurp* knockout, HJURP loss in p53-null transformed cells and tumors led to altered ploidy and cell death via apoptosis (Figs. 4, 6). Our hypothesis is thus that centromere composition might actually be sensed directly or indirectly by p53 to ensure the integrity of this key chromosomal landmark. We thus propose a model in which, in p53-proficient cells undergoing normal proliferation, HJURP knockout results in a slow depletion of CENP-A, which is sensed by p53. p53 activation subsequently blocks cell cycle progression in time to prevent mitosis from ensuing in cells with incompetent centromeres, thus safeguarding genome integrity (see the proposed model in Fig. 8). Our model predicts that two aspects contribute to the critical susceptibility of transformed cells to HJURP knockout, as demonstrated in our p53-null mouse tumors (Fig. 7). On the one hand, their increased proliferation rate, which imposes kinetic constraints on CENP-A deposition, results in the rapid loss of CENP-A from centromeres, as it is diluted twofold every cell division in the absence of new deposition (Fachinetti et al. 2013). On the other hand, loss of p53 function results in the unchecked entry into mitosis of cells whose centromeres cannot support proper chromosome segregation, thus leading to aneuploidy and apoptosis. Chromosomal missegregation and aneuploidy can both interfere with and promote tumorigenesis (Santaguida and Amon 2015). However, given the role of HJURP in depositing CENP-A at all centromeres, depleting it affects all chromosomes universally, and the massive aneuploidy that ensues has a net detrimental effect on the tumor. These aspects of chromosome dynamics add a further dimension to the role of HJURP in tumorigenesis, and the potential contribution of aneuploidy to blocking tumorigenesis in the context of therapeutically targeting HJURP thus merits further attention.

The fact that HJURP and CENP-A are up-regulated as a consequence of transformation and that p53-null cells subsequently become addicted to increased levels of these centromeric factors suggests roles that are clearly distinct from classic drivers of transformation. Importantly, the role of the CENP-A histone variant in tumorigenesis should be distinguished from that of oncohistones, which contain K27M or K36M mutations. These mutations alter differentiation pathways in particular cell types, leading to tissue-specific cancers (Schwartzentruber et al. 2012; Sturm et al. 2012; Fang et al. 2016; Lu et al. 2016). On the other hand, CENP-A is critical for proliferation in all cell types. We thus propose that HJURP acts as a universal “navigator” that keeps the cancer cell on its path of hyperproliferation and potentiates further progressive changes associated with tumor development. Our study thus supports a role for HJURP and CENP-A in sustaining cellular transformation. It is tempting to speculate that inhibiting CENP-A deposition will impede progression in an existing tumor while inducing milder consequences in surrounding healthy tissue that has both a lower rate of proliferation and a functional p53 response. This “epigenetic addiction” would thus underscore the therapeutic potential of targeting CENP-A deposition or centromere organization as an Achilles’ heel in p53-defective tumors.

## Materials and methods

### Data analysis (TCGA data)

Exome and RNA sequencing data sets from TCGA (http://tcga-data.nci.nih.gov/tcga) were analyzed in TCGA provisional data sets for all cancers (28 cancer types), which included adrenocortical carcinoma; acute myeloid leukemia; bladder urothelial carcinoma; breast-invasive carcinoma; cervical squamous cell carcinoma; endocervical adenocarcinoma; cholangiocarcinoma; colorectal adenocarcinoma; lymphoid neoplasm diffuse large B-cell lymphoma; glioblastoma multiforme; brain lower-grade glioma; head and neck squamous cell carcinoma; liver hepatocellular carcinoma; lung adenocarcinoma; lung squamous cell carcinoma; skin cutaneous melanoma; ovarian serous cystadenocarcinoma, pheochromocytoma, and paraganglioma; pancreatic adenocarcinoma; prostate adenocarcinoma; sarcoma; testicular germ cell cancer; thyroid carcinoma; uterine corpus endometrial carcinoma; uterine carcinosarcoma; uveal melanoma; kidney renal clear cell carcinoma; kidney chromophobe; and kidney renal papillary cell carcinoma. In separate analyses, breast cancers (Ciriello et al. 2015), melanoma (TCGA provisional data), and pancreatic cancers (TCGA provisional data) were studied. In each case, tumors were grouped according to *TP53* mutational status: wild-type *TP53* (diploid with no detectable mutations) and *TP53* loss-of-function tumors (either homozygous deletion or heterozygous deletion and nonsense mutation or in-frame truncations of the second allele). All other *TP53* mutations whose effect could not be inferred from genetic status alone were excluded. A Wilcoxon rank sum test was used to assess statistical significance.

### Plasmids and siRNA

The retroviral vectors pWZL (hygro), pWZL-E1A (hygro), pWZL-HRas-V12 (neo) (Lowe et al. 1994), and Ras-IRES-blast and pLEX vectors were a gift from Dr. Laura D. Attardi. The HRas-V12 insert was transferred from pWZL-HRas-V12 (neo) into pWZL (hygro) linearized with BamHI and SalI. Mouse *Hjurp* cDNA (accession no. NM_198652.2; synthesized by GenScript) and *Cenpa* cDNA (accession no. NM_007681.2; purchased from Open Biosystems) were cloned into the BamHI and SalI sites of pWZL. C102T and G105A silent mutations were introduced into pWZL-Hjurp, and then the Hjurp-Hygro gene cassette was transferred into the pLVX-TET-One vector (Clontech) using Infusion cloning (Clontech). gRNA sequences targeting GFP (5′- GAGCTGGACGGCGACGTAAA-3′) and *Hjurp* (#1, 5′-AAGCGGCTGATAGCGAAGGT-3′; and #2, 5′-ACGGGTCGTCCTCAAAGGGC-3′) were cloned into the lentiCRISPR v2 plasmid (Sanjana et al. 2014). These sequences target the exon 1–intron boundary and exon 2 of *Hjurp*, respectively (Supplemental Fig. S3C). LentiCRISPR v2 plasmids containing gRNA sequences against human *HJURP* (#3, 5′-GGTCGATGCCACGTCAGACC-3′; and #4, 5′-TCCCTCGCACCGCACAGTCC-3′) were obtained from Genescript. siRNA against *Cenpa* (#1, 5′-CACAGUCGGCGGAGACAAGtt-3′; and #2, 5′-CUCGUGGUGUGGACUUCAAtt-3′) and control siRNA against luciferase (Luc, 5′-UGGACAAUUAUGGACAACA-3′) were obtained from MWG Eurofins.

### HJURP antibody production

His-tagged mouse HJURP was expressed from the pET30a vector in the Rosetta *Escherichia coli* strain (Novagen). The recombinant protein was present exclusively in inclusion bodies, which were washed twice by sonication in 2 M urea and completely solubilized in 6 M urea. This solution was used for subcutaneous immunization in two rabbits (Agro-Bio). The serum showing the best response was used at 1:2000 in Western blots.

### Gel electrophoresis, immunoblotting, and antibodies

The NuPAGE electrophoresis system was used with NuPAGE 4%–12% Bis-Tris gradient gels and MOPS buffer (Invitrogen). For transfers, the Trans-blot Turbo system (Bio-Rad) was used with PVDF transfer packs. For Western blotting, total extracts were prepared by resuspending cell pellets in RIPA buffer (150 mM NaCl, 1% NP-40, 50 mM Tris-HCl at pH 8, 1% SDS) supplemented with 1:100 benzonase (Novagen) and 1× Complete protease inhibitor (CPI) cocktail (Roche) followed by sonication in a water bath. Extracts were prepared from tumors in the same way after tissue disruption using a Dounce homogenizer. Protein concentration was determined by the Lowry method (Bio-Rad DC protein assay). Serial twofold dilutions (40, 20, and 10 µg) of each sample were loaded for all gels. Antibodies used in this study were as follows: mouse CENP-A (Cell Signaling no. 2048 for immunofluorescence or Abcam no. ab33565 for Western), human CENP-A (Epitomics, no. 1745-1), mouse HJURP polyclonal antibody (generated in-house), human HJURP (Sigma, HPA008436), CAF-1 p150 polyclonal antibody (generated in-house), β-actin (Sigma, A1978), γ-tubulin (Sigma, T5326), α-tubulin (Sigma, T9026), Histone H3 (Abcam, ab1791), Histone H4 (Abcam, ab31830), BrdU (clone BU1/75 [ICR1], AbD Serotec), cleaved caspase-3 Asp175 (Cell Signaling, no. 9661), Histone γH2A.X (Millipore, no. 05-636), p53 (Leica Biosystems, CM5 Novocastra), P-Rb S807/811 (Cell Signaling, no. 8516), P-cdc2 (Y15) (Cell Signaling, no. 9111), and p-Chk1 S345 (Cell Signaling, no. 2348). Total protein was detected with Memcode reversible protein stain (Thermo scientific).

### Luciferase expression assays

Luciferase reporter plasmids were constructed by cloning a 2-kb-long fragment centered around the TSS upstream of the firefly luciferase gene in a pGL3-basic vector (Promega) for each promoter or a variant fragment generated by PCR mutagenesis of the putative CDE/CHR motif. NIH/3T3 cells (10^6^ cells) were transfected using Lipofectamine 2000 with 3 µg of the luciferase reporter plasmid and 30 ng of Renilla luciferase expression plasmid (pGL4.73; Promega) for normalization and treated or not with 10 µM nutlin 3a. Transfected cells were incubated for 24 h, trypsinized, resuspended in 75 µL of culture medium with 7.5% fetal calf serum, and transferred into a well of an optical 96-well plate (Nunc). The Dual-glo luciferase assay system (Promega) was used according to the manufacturer's protocol to lyse the cells and read firefly and Renilla luciferase signals. Results were normalized, and the average luciferase activity in cells transfected with a wild-type promoter and not treated with nutlin was assigned a value of 1. Differences between two groups were analyzed by Student's *t*-test, and values of *P* ≤ 0.05 were considered significant.

### Immunofluorescence and DNA FISH

To prepare metaphase spreads, cells were treated with 60 ng/mL nocodazole (Sigma) overnight, and mitotic cells were harvested by shake-off, resuspended at 10^6^ cells per milliliter in 75 mM KCl 10 mM HEPES (pH 8), and allowed to swell for 30 min at room temperature. After dilution to 2 × 10^5^ cells per milliliter, 250 µL was spun onto coverslips in a Cytospin 3 centrifuge (Thermo Scientific) at 2000 rpm for 10 min. CENP-A was immunofluorescently labeled prior to fixation in KCM buffer (120 mM KCI, 20 mM NaCl, 10 mM Tris-HCl at pH 8, 0.5 mM EDTA, 0.1% [v/v] Triton X-100, protease inhibitors) as described in Guenatri et al. (2004). After fixation in KCM containing 1:10 formalin (∼3.7% formaldehyde final) for 10 min, DNA FISH was performed against minor and major satellites using LNA probes (Probst et al. 2010) or probes generated by nick translation from BACs using ARES Alexa fluor kits (Thermo Scientific). For metaphase spreads, images were acquired on a LSM 780 confocal microscope using a 63× immersion objective, 2× internal magnification, and optimal voxel size and controlled by ZEN software (Carl Zeiss). Maximum intensity projections of 10 0.39-µm Z slices were generated using ImageJ. For tumor sections, images were acquired on a Zeiss Z1 epifluorescence microscope using a 100× immersion objective or on an upright Leica DM6000 using HCX plan apo 40× or 100× oil objectives. Single-plane images were used for CENP-A counts, or maximum intensity projections of 30–40 0.2-µm Z slices were generated using ImageJ.

### Gene expression analysis

RNA was extracted from cells using an RNeasy kit with QIAshredder disruption and on-column DNase digestion automated on a QIAcube device (Qiagen) or using Nucleospin RNA II (Macherey-Nagel). Reverse transcription and qPCR were carried out as described previously (Boyarchuk et al. 2014). Real-time qPCRs were performed on an ABI PRISM 7500 using Power SYBR Green (Applied Biosystems). Corresponding qPCR primers are summarized in Supplemental Tables 1 and 2. Differences between two groups were analyzed by Student's *t*-test, difference between three groups were analyzed by one-way ANOVA, and values of *P* ≤ 0.05 were considered significant.

### Proliferation and substrate-independent growth assays

For growth curves, 3 × 10^4^ MEFs were seeded in 1 mL of growth medium on 24-well plates in triplicate for each experimental point. Viable cell numbers were counted after harvesting with trypsin on a Vi-CELL XR counter (Beckman Coulter). Soft agar assays were performed as in Kenzelmann Broz et al. (2013), but cell number was reduced to 2 × 10^4^ for p53-null MEFs transformed with E1A + HRas-V12. Three weeks or 4 wk later, colonies were stained with 5 nM Sytox Green (Thermo Scientific) and imaged on a Typhoon FLA 9500 instrument (GE Healthcare).

### MEF derivation and cell culture

p53 heterozygous animals (Donehower et al. 1992) backcrossed into the C57BL6/N genetic background were mated to obtain p53^+/+^ (wild-type) and p53^−/−^ (null) embryos. MEFs isolated from day 13.5 embryos were cultured for six or fewer passages in a 5% CO_2_ and 3% O_2_ incubator in DMEM (Gibco) supplemented with 10% fetal calf serum and penicillin/streptomycin (Life Technologies) in 5% CO_2_ and 3% O_2_. Once oncogenically transformed with E1A or HRas-V12 oncogenes, MEFs were cultured in atmospheric O_2_ concentration. MEFs described in Figure 2 (p53-null, wild-type, or p53^Δ31/Δ31^ [Δ31]) were maintained as above in DMEM Glutamax (Gibco) with 15% fetal calf serum, 100 µM 2-mercaptoethanol (Millipore), and 10 µM nonessential amino acids and penicillin/streptomycin (NEAA/PS) (Gibco). Human lung fibroblasts MRC5 and their SV40 transformed derivatives (MRC5 SV2; Sigma) were maintained as above in MEM (Gibco) completed with 10% fetal calf serum, 2 mM L-glutamine (Gibco), 1 mM pyruvate, and 10 µM NEAA/PS. Cells were treated with 10 µM nutlin 3a for 24 h before RT–PCR or luciferase assays. MCF7 parental control and p53 DD cell lines were a gift from Moshe Oren (Weizmann Institute, Israel). All other cell lines were maintained in DMEM (Gibco) supplemented with 10% fetal calf serum and penicillin/streptomycin (Life Technologies).

### Viral transduction

Ecotropic retroviral particles were generated by transfecting pWZL vectors into the Phoenix-Eco packaging cell line using JetPRIME (Polyplus). Pseudotyped lentiviral particles were generated with Ras-IRES-blast, LentiCRISPR v2, and pLVX-TetOne vectors by cotransfection with psPAX2 and pMD2.G vectors at a ratio of 4:3:1 into 293T cells. Infection was carried out in the presence of 8 µg/mL polybrene (Sigma). Twenty-four hours later, transduced cells were selected with 2 µg/mL puromycin for LentiCRISPR v2, 200 µg/mL hygromycin for pWZL and pLVX-TetOne-HJURP, and 10 µg/mL blasticidin for Ras-IRES-blast (drugs purchased from Life Technologies) and maintained in the presence of drugs permanently. Cells were used for experiments or further rounds of transduction after a minimum of 4 d of selection. Serial transductions were carried out in the following order: pWZL followed by TetOne-HJURP or Ras-IRES-blast and then LentiCRISPR v2 vectors.

### Flow cytometry

Cell cycle was analyzed using a Click-iT EdU Alexa fluor 647 flow cytometry assay kit (Life Technologies) according to the manufacturer's instructions. MEFs were incubated with 10 µM EdU for 2–4 h, depending on their proliferation rate. Apoptosis was analyzed using an Alexa fluor 488-Annexin V/dead cell apoptosis kit (Life Technologies). Labeled cells were analyzed on Accuri C6 and LSR II flow cytometers (BD Biosciences). For cell-sorting experiments, NIH/3T3 cells were treated with 10 µg/mL Hoechst 33342 for 30 min and then sorted according to DNA content on a FACSAria III equipped with a 130-µm nozzle (BD Biosciences) at the Curie Institute Flow Cytometry platform. Data were processed using FlowJo X software (Tree Star).

### Mouse genome analysis

Mouse genome paired-end resequencing data were obtained from the Sanger Institute Mouse Genomes Project (http://www.sanger.ac.uk/resources/mouse/genomes). An analysis of the strains C57BL/6NJ (ERS076384), BALB/cJ (ERS076386), CAST/EiJ (ERS076381), and SPRET/EiJ (ERS076388 and ERS138732) was conducted. These data had already been aligned to the mm9 reference genome. Copy number variations were detected using Control-FREEC (Boeva et al. 2011, 2012) with a window and step size of 5000 and 1000 base pairs, respectively, and an assumed ploidy of 2. All other parameters were kept at default. In order to get a higher-resolution view of the Hjurp gene, the read depth per base was calculated using the Genomecov component of the Bedtools suite (Quinlan and Hall 2010). The sequence of Hjurp intron 4 was downloaded from ENSEMBL and then submitted to RepeatMasker (Tarailo-Graovac and Chen 2009) in order to detect any repeats within. Positions of the repeat regions were inferred from the masked sequence output.

### Subcutaneous tumor model

Animals were used in accordance with the International Guiding Principles for Biomedical Research Involving Animals as promulgated by the Society for the Study of Reproduction and the European Convention on Animal Experimentation. p53-null HRas-V12 transformed MEFs were sequentially transduced with CRISPR-resistant doxycycline-inducible HJURP followed by Cas9–sgRNA constructs against either GFP (control) or one of two sites of Hjurp (see Fig. 5A). Following at least 6 d under puromycin selection and HJURP transgene induction with doxycycline, 10^6^ cells in 100 µL of Opti-MEM were injected into the anterior flanks of 6-wk-old female BALB/c nude mice (Charles River). Three mice were injected bilaterally for each CRISPR construct. Doxycycline was administered at 2 mg/mL as a loading dose 24 h before cell injection and then reduced to 0.2 mg/mL 24 h after injection to avoid accumulation in tissues. Solutions were prepared fresh every other day in drinking water supplemented with 5% sucrose. Following doxycycline withdrawal 6 or 10 d after injection, 5% sucrose was maintained in drinking water. Tumor volume was measured as described (Brady et al. 2011). One-hundred micrograms of BrdU (Sigma) per gram of body weight was injected intraperitoneally 1 h before sacrificing the animals. Tumors were then harvested for Western blot analysis or fixed in PFA and embedded in paraffin, and sections were processed for immunofluorescence analysis. Statistical analyses were computed in Prism7 using *t*-tests.

## Supplementary Material

Supplemental Material

## References

[FILIPESCUGAD290924C1] Allshire RC, Karpen GH. 2008 Epigenetic regulation of centromeric chromatin: old dogs, new tricks? Nat Rev Genet 9: 923–937. 19002142 10.1038/nrg2466 PMC2586333

[FILIPESCUGAD290924C2] Athwal RK, Walkiewicz MP, Baek S, Fu S, Bui M, Camps J, Ried T, Sung M-H, Dalal Y. 2015 CENP-A nucleosomes localize to transcription factor hotspots and subtelomeric sites in human cancer cells. Epigenetics Chromatin 8: 2. 25788983 10.1186/1756-8935-8-2 PMC4363203

[FILIPESCUGAD290924C3] Beckerman R, Prives C. 2010 Transcriptional regulation by p53. Cold Spring Harb Perspect Biol 2: a000935. 20679336 10.1101/cshperspect.a000935 PMC2908772

[FILIPESCUGAD290924C4] Benson EK, Mungamuri SK, Attie O, Kracikova M, Sachidanandam R, Manfredi JJ, Aaronson SA. 2014 p53-dependent gene repression through p21 is mediated by recruitment of E2F4 repression complexes. Oncogene 33: 3959–3969. 24096481 10.1038/onc.2013.378 PMC4067464

[FILIPESCUGAD290924C5] Bieging KT, Mello SS, Attardi LD. 2014 Unravelling mechanisms of p53-mediated tumour suppression. Nat Rev Cancer 14: 359–370. 24739573 10.1038/nrc3711 PMC4049238

[FILIPESCUGAD290924C6] Black BE, Jansen LET, Foltz DR, Cleveland DW. 2010 Centromere identity, function, and epigenetic propagation across cell divisions. Cold Spring Harb Symp Quant Biol 75: 403–418. 21467140 10.1101/sqb.2010.75.038 PMC3140419

[FILIPESCUGAD290924C7] Boeva V, Zinovyev A, Bleakley K, Vert J-P, Janoueix-Lerosey I, Delattre O, Barillot E. 2011 Control-free calling of copy number alterations in deep-sequencing data using GC-content normalization. Bioinformatics 27: 268–269. 21081509 10.1093/bioinformatics/btq635 PMC3018818

[FILIPESCUGAD290924C8] Boeva V, Popova T, Bleakley K, Chiche P, Cappo J, Schleiermacher G, Janoueix-Lerosey I, Delattre O, Barillot E. 2012 Control-FREEC: a tool for assessing copy number and allelic content using next-generation sequencing data. Bioinformatics 28: 423–425. 22155870 10.1093/bioinformatics/btr670 PMC3268243

[FILIPESCUGAD290924C9] Boyarchuk E, Filipescu D, Vassias I, Cantaloube S, Almouzni G. 2014 The histone variant composition of centromeres is controlled by the pericentric heterochromatin state during the cell cycle. J Cell Sci 127: 3347–3359. 24906798 10.1242/jcs.148189

[FILIPESCUGAD290924C10] Brady CA, Jiang D, Mello SS, Johnson TM, Jarvis LA, Kozak MM, Kenzelmann Broz D, Basak S, Park EJ, McLaughlin ME, 2011 Distinct p53 transcriptional programs dictate acute DNA-damage responses and tumor suppression. Cell 145: 571–583. 21565614 10.1016/j.cell.2011.03.035 PMC3259909

[FILIPESCUGAD290924C11] Carone DM, Lawrence JB. 2013 Heterochromatin instability in cancer: from the Barr body to satellites and the nuclear periphery. Semin Cancer Biol 23: 99–108. 22722067 10.1016/j.semcancer.2012.06.008 PMC3500402

[FILIPESCUGAD290924C112] Ciriello G, Gatza ML, Beck AH, Wilkerson MD, Rhie SK, Pastore A, Zhang H, McLellan M, Yau C, Kandoth C, et al. 2015 Comprehensive molecular portraits of invasive lobular breast cancer. Cell 163: 506–519. 26451490 10.1016/j.cell.2015.09.033 PMC4603750

[FILIPESCUGAD290924C12] de Tayrac M, Saïkali S, Aubry M, Bellaud P, Boniface R, Quillien V, Mosser J. 2013 Prognostic significance of EDN/RB, HJURP, p60/CAF-1 and PDLI4, four new markers in high-grade gliomas. PLoS One 8: e73332. 24039914 10.1371/journal.pone.0073332 PMC3770632

[FILIPESCUGAD290924C14] Donehower LA, Harvey M, Slagle BL, McArthur MJ, Montgomery CA, Butel JS, Bradley A. 1992 Mice deficient for p53 are developmentally normal but susceptible to spontaneous tumours. Nature 356: 215–221. 1552940 10.1038/356215a0

[FILIPESCUGAD290924C15] Dunleavy EM, Roche D, Tagami H, Lacoste N, Ray-Gallet D, Nakamura Y, Daigo Y, Nakatani Y, Almouzni-Pettinotti G. 2009 HJURP is a cell-cycle-dependent maintenance and deposition factor of CENP-A at centromeres. Cell 137: 485–497. 19410545 10.1016/j.cell.2009.02.040

[FILIPESCUGAD290924C16] Earnshaw WC, Rothfield N. 1985 Identification of a family of human centromere proteins using autoimmune sera from patients with scleroderma. Chromosoma 91: 313–321. 2579778 10.1007/BF00328227

[FILIPESCUGAD290924C17] Fachinetti D, Folco HD, Nechemia-Arbely Y, Valente LP, Nguyen K, Wong AJ, Zhu Q, Holland AJ, Desai A, Jansen LET, 2013 A two-step mechanism for epigenetic specification of centromere identity and function. Nat Cell Biol 15: 1056–1066. 23873148 10.1038/ncb2805 PMC4418506

[FILIPESCUGAD290924C18] Fang D, Gan H, Lee J-H, Han J, Wang Z, Riester SM, Jin L, Chen J, Zhou H, Wang J, 2016 The histone H3.3K36M mutation reprograms the epigenome of chondroblastomas. Science 352: 1344–1348. 27229140 10.1126/science.aae0065 PMC5460624

[FILIPESCUGAD290924C19] Filipescu D, Müller S, Almouzni G. 2014 Histone H3 variants and their chaperones during development and disease: contributing to epigenetic control. Annu Rev Cell Dev Biol 30: 615–646. 25288118 10.1146/annurev-cellbio-100913-013311

[FILIPESCUGAD290924C20] Fischer M, Grossmann P, Padi M, DeCaprio JA. 2016 Integration of TP53, DREAM, MMB–FOXM1 and RB–E2F target gene analyses identifies cell cycle gene regulatory networks. Nucleic Acids Res 44: 6070–6086. 27280975 10.1093/nar/gkw523 PMC4994865

[FILIPESCUGAD290924C21] Foltz DR, Jansen LET, Bailey AO, Yates JR, Bassett EA, Wood S, Black BE, Cleveland DW. 2009 Centromere-specific assembly of CENP-a nucleosomes is mediated by HJURP. Cell 137: 472–484. 19410544 10.1016/j.cell.2009.02.039 PMC2747366

[FILIPESCUGAD290924C22] Ghiselli G. 2006 SMC3 knockdown triggers genomic instability and p53-dependent apoptosis in human and zebrafish cells. Mol Cancer 5: 52. 17081288 10.1186/1476-4598-5-52 PMC1636066

[FILIPESCUGAD290924C23] Grant GD, Brooks L, Zhang X, Mahoney JM, Martyanov V, Wood TA, Sherlock G, Cheng C, Whitfield ML. 2013 Identification of cell cycle-regulated genes periodically expressed in U2OS cells and their regulation by FOXM1 and E2F transcription factors. Mol Biol Cell 24: 3634–3650. 24109597 10.1091/mbc.E13-05-0264 PMC3842991

[FILIPESCUGAD290924C24] Gu X-M, Fu J, Feng X-J, Huang X, Wang S-M, Chen X-F, Zhu M-H, Zhang S-H. 2014 Expression and prognostic relevance of centromere protein A in primary osteosarcoma. Pathol Res Pract 210: 228–233. 24440098 10.1016/j.prp.2013.12.007

[FILIPESCUGAD290924C25] Guenatri M, Bailly D, Maison C, Almouzni G. 2004 Mouse centric and pericentric satellite repeats form distinct functional heterochromatin. J Cell Biol 166: 493–505. 15302854 10.1083/jcb.200403109 PMC2172221

[FILIPESCUGAD290924C26] Hahn WC, Dessain SK, Brooks MW, King JE, Elenbaas B, Sabatini DM, DeCaprio JA, Weinberg RA. 2002 Enumeration of the simian virus 40 early region elements necessary for human cell transformation. Mol Cell Biol 22: 2111–2123. 11884599 10.1128/MCB.22.7.2111-2123.2002 PMC133688

[FILIPESCUGAD290924C27] Harvey M, Sands AT, Weiss RS, Hegi ME, Wiseman RW, Pantazis P, Giovanella BC, Tainsky MA, Bradley A, Donehower LA. 1993 In vitro growth characteristics of embryo fibroblasts isolated from p53-deficient mice. Oncogene 8: 2457–2467. 8103211

[FILIPESCUGAD290924C28] Heo J-I, Cho JH, Kim J-R. 2013 HJURP regulates cellular senescence in human fibroblasts and endothelial cells via a p53-dependent pathway. J Gerontol A Biol Sci Med Sci 68: 914–925. 23292286 10.1093/gerona/gls257

[FILIPESCUGAD290924C29] Hoffmann S, Dumont M, Barra V, Ly P, Nechemia-Arbely Y, McMahon MA, Hervé S, Cleveland DW, Fachinetti D. 2016 CENP-A is dispensable for mitotic centromere function after initial centromere/kinetochore assembly. Cell Rep 17: 2394–2404. 27880912 10.1016/j.celrep.2016.10.084 PMC5134894

[FILIPESCUGAD290924C30] Howman EV, Fowler KJ, Newson AJ, Redward S, MacDonald AC, Kalitsis P, Choo KH. 2000 Early disruption of centromeric chromatin organization in centromere protein A (Cenpa) null mice. Proc Natl Acad Sci 97: 1148–1153. 10655499 10.1073/pnas.97.3.1148 PMC15551

[FILIPESCUGAD290924C31] Hu Z, Huang G, Sadanandam A, Gu S, Lenburg ME, Pai M, Bayani N, Blakely EA, Gray JW, Mao J-H. 2010 The expression level of HJURP has an independent prognostic impact and predicts the sensitivity to radiotherapy in breast cancer. Breast Cancer Res 12: R18. 20211017 10.1186/bcr2487 PMC2879562

[FILIPESCUGAD290924C32] Jaber S, Toufektchan E, Lejour V, Bardot B, Toledo F. 2016 p53 downregulates the Fanconi anaemia DNA repair pathway. Nat Commun 7: 11091. 27033104 10.1038/ncomms11091 PMC4821997

[FILIPESCUGAD290924C33] Jansen LET, Black BE, Foltz DR, Cleveland DW. 2007 Propagation of centromeric chromatin requires exit from mitosis. J Cell Biol 176: 795–805. 17339380 10.1083/jcb.200701066 PMC2064054

[FILIPESCUGAD290924C34] Kato T, Sato N, Hayama S, Yamabuki T, Ito T, Miyamoto M, Kondo S, Nakamura Y, Daigo Y. 2007 Activation of Holliday junction recognizing protein involved in the chromosomal stability and immortality of cancer cells. Cancer Res 67: 8544–8553. 17823411 10.1158/0008-5472.CAN-07-1307

[FILIPESCUGAD290924C35] Kaufman PD, Kobayashi R, Kessler N, Stillman B. 1995 The p150 and p60 subunits of chromatin assembly factor I: a molecular link between newly synthesized histones and DNA replication. 81: 1105–1114. 10.1016/s0092-8674(05)80015-7 7600578

[FILIPESCUGAD290924C36] Keane TM, Goodstadt L, Danecek P, White MA, Wong K, Yalcin B, Heger A, Agam A, Slater G, Goodson M, 2011 Mouse genomic variation and its effect on phenotypes and gene regulation. Nature 477: 289–294. 21921910 10.1038/nature10413 PMC3276836

[FILIPESCUGAD290924C37] Kenzelmann Broz D, Spano Mello S, Bieging KT, Jiang D, Dusek RL, Brady CA, Sidow A, Attardi LD. 2013 Global genomic profiling reveals an extensive p53-regulated autophagy program contributing to key p53 responses. Genes Dev 27: 1016–1031. 23651856 10.1101/gad.212282.112 PMC3656320

[FILIPESCUGAD290924C38] Kim E-H, Lee Y-J, Bae S, Lee JS, Kim J, Lee Y-S. 2009 Heat shock factor 1-mediated aneuploidy requires a defective function of p53. Cancer Res 69: 9404–9412. 19934326 10.1158/0008-5472.CAN-09-1411

[FILIPESCUGAD290924C39] Lacoste N, Woolfe A, Tachiwana H, Garea AV, Barth T, Cantaloube S, Kurumizaka H, Imhof A, Almouzni G. 2014 Mislocalization of the centromeric histone variant CenH3/CENP-A in human cells depends on the chaperone DAXX. Mol Cell 53: 631–644. 24530302 10.1016/j.molcel.2014.01.018

[FILIPESCUGAD290924C40] Lambrus BG, Uetake Y, Clutario KM, Daggubati V, Snyder M, Sluder G, Holland AJ. 2015 p53 protects against genome instability following centriole duplication failure. J Cell Biol 210: 63–77. 26150389 10.1083/jcb.201502089 PMC4494000

[FILIPESCUGAD290924C41] Leibiger C, Kosyakova N, Mkrtchyan H, Glei M, Trifonov V, Liehr T. 2013 First molecular cytogenetic high resolution characterization of the NIH 3T3 cell line by murine multicolor banding. J Histochem Cytochem 61: 306–312. 23321776 10.1369/0022155413476868 PMC3621507

[FILIPESCUGAD290924C42] Li J, Anderson MG, Tucker LA, Shen Y, Glaser KB, Shah OJ. 2009 Inhibition of Aurora B kinase sensitizes a subset of human glioma cells to TRAIL concomitant with induction of TRAIL-R2. Cell Death Differ 16: 498–511. 19079141 10.1038/cdd.2008.174

[FILIPESCUGAD290924C43] Li Y, Zhu Z, Zhang S, Yu D, Yu H, Liu L, Cao X, Wang L, Gao H, Zhu M. 2011 shRNA-targeted centromere protein A inhibits hepatocellular carcinoma growth. PLoS One 6: e17794. 21423629 10.1371/journal.pone.0017794 PMC3058037

[FILIPESCUGAD290924C144] Liu S-T, Yen TJ. 2009 The kinetochore as target for cancer drug development. In The kinetochore: from molecular discoveries to cancer therapy (ed. De Wulf P, Earnshaw WC), pp. 455–479. Springer, New York.

[FILIPESCUGAD290924C44] Löhr K, Möritz C, Contente A, Dobbelstein M. 2003 p21/CDKN1A mediates negative regulation of transcription by p53. J Biol Chem 278: 32507–32516. 12748190 10.1074/jbc.M212517200

[FILIPESCUGAD290924C45] Lowe SW, Jacks T, Housman DE, Ruley HE. 1994 Abrogation of oncogene-associated apoptosis allows transformation of p53-deficient cells. Proc Natl Acad Sci 91: 2026–2030. 8134344 10.1073/pnas.91.6.2026 PMC43302

[FILIPESCUGAD290924C46] Lu C, Jain SU, Hoelper D, Bechet D, Molden RC, Ran L, Murphy D, Venneti S, Hameed M, Pawel BR, 2016 Histone H3K36 mutations promote sarcomagenesis through altered histone methylation landscape. Science 352: 844–849. 27174990 10.1126/science.aac7272 PMC4928577

[FILIPESCUGAD290924C47] Luo J, Solimini NL, Elledge SJ. 2009 Principles of cancer therapy: oncogene and non-oncogene addiction. Cell 136: 823–837. 19269363 10.1016/j.cell.2009.02.024 PMC2894612

[FILIPESCUGAD290924C48] Ma X-J, Salunga R, Tuggle JT, Gaudet J, Enright E, McQuary P, Payette T, Pistone M, Stecker K, Zhang BM, 2003 Gene expression profiles of human breast cancer progression. Proc Natl Acad Sci 100: 5974–5979. 12714683 10.1073/pnas.0931261100 PMC156311

[FILIPESCUGAD290924C49] Maehara K, Takahashi K, Saitoh S. 2010 CENP-A reduction induces a p53-dependent cellular senescence response to protect cells from executing defective mitoses. Mol Cell Biol 30: 2090–2104. 20160010 10.1128/MCB.01318-09 PMC2863584

[FILIPESCUGAD290924C50] Mahadevan D, Morales C, Cooke LS, Manziello A, Mount DW, Persky DO, Fisher RI, Miller TP, Qi W. 2014 Alisertib added to rituximab and vincristine is synthetic lethal and potentially curative in mice with aggressive DLBCL co-overexpressing MYC and BCL2. PLoS One 9: e95184. 24893165 10.1371/journal.pone.0095184 PMC4043492

[FILIPESCUGAD290924C51] McKinley KL, Cheeseman IM. 2016 The molecular basis for centromere identity and function. Nat Rev Mol Cell Biol 17: 16–29. 26601620 10.1038/nrm.2015.5 PMC8603311

[FILIPESCUGAD290924C52] Montes de Oca R, Gurard-Levin ZA, Berger F, Rehman H, Martel E, Corpet A, De Koning L, Vassias I, Wilson LOW, Meseure D, 2015 The histone chaperone HJURP is a new independent prognostic marker for luminal A breast carcinoma. Mol Oncol 9: 657–674. 25497280 10.1016/j.molonc.2014.11.002 PMC5528705

[FILIPESCUGAD290924C53] Müller S, Almouzni G. 2014 A network of players in H3 histone variant deposition and maintenance at centromeres. Biochim Biophys Acta 1839: 241–250. 24316467 10.1016/j.bbagrm.2013.11.008

[FILIPESCUGAD290924C54] Müller S, Almouzni G. 2017 Chromatin dynamics during the cell cycle at centromeres. Nat Rev Genet 18: 192–208. 28138144 10.1038/nrg.2016.157

[FILIPESCUGAD290924C55] Müller S, Montes de Oca R, Lacoste N, Dingli F, Loew D, Almouzni G. 2014 Phosphorylation and DNA binding of HJURP determine its centromeric recruitment and function in CenH3(CENP-A) loading. Cell Rep 8: 190–203. 25001279 10.1016/j.celrep.2014.06.002

[FILIPESCUGAD290924C56] Murphy KL, Rosen JM. 2000 Mutant p53 and genomic instability in a transgenic mouse model of breast cancer. Oncogene 19: 1045–1051. 10713688 10.1038/sj.onc.1203274

[FILIPESCUGAD290924C57] Ohashi A, Ohori M, Iwai K, Nakayama Y, Nambu T, Morishita D, Kawamoto T, Miyamoto M, Hirayama T, Okaniwa M, 2015 Aneuploidy generates proteotoxic stress and DNA damage concurrently with p53-mediated post-mitotic apoptosis in SAC-impaired cells. Nat Commun 6: 7668. 26144554 10.1038/ncomms8668 PMC4506520

[FILIPESCUGAD290924C58] Pezer Ž, Harr B, Teschke M, Babiker H, Tautz D. 2015 Divergence patterns of genic copy number variation in natural populations of the house mouse (*Mus musculus domesticus*) reveal three conserved genes with major population-specific expansions. Genome Res 25: 1114–1124. 26149421 10.1101/gr.187187.114 PMC4509996

[FILIPESCUGAD290924C59] Polo SE, Theocharis SE, Klijanienko J, Savignoni A, Asselain B, Vielh P, Almouzni G. 2004 Chromatin assembly factor-1, a marker of clinical value to distinguish quiescent from proliferating cells. Cancer Res 64: 2371–2381. 15059888 10.1158/0008-5472.can-03-2893

[FILIPESCUGAD290924C160] Polo SE, Theocharis SE, Grandin L, Gambotti L, Antoni G, Savignoni A, Asselain B, Patsouris E, Almouzni G. 2010 Clinical significance and prognostic value of chromatin assembly factor-1 overexpression in human solid tumours. Histopathology 57: 716–724. 21083601 10.1111/j.1365-2559.2010.03681.x

[FILIPESCUGAD290924C60] Probst AV, Okamoto I, Casanova M, Marjou El F, Le Baccon P, Almouzni G. 2010 A strand-specific burst in transcription of pericentric satellites is required for chromocenter formation and early mouse development. Dev Cell 19: 625–638. 20951352 10.1016/j.devcel.2010.09.002

[FILIPESCUGAD290924C61] Qiu J-J, Guo J-J, Lv T-J, Jin H-Y, Ding J-X, Feng W-W, Zhang Y, Hua K-Q. 2013 Prognostic value of centromere protein-A expression in patients with epithelial ovarian cancer. Tumour Biol 34: 2971–2975. 23712606 10.1007/s13277-013-0860-6

[FILIPESCUGAD290924C62] Quaas M, Müller GA, Engeland K. 2012 p53 can repress transcription of cell cycle genes through a p21(WAF1/CIP1)-dependent switch from MMB to DREAM protein complex binding at CHR promoter elements. Cell Cycle 11: 4661–4672. 23187802 10.4161/cc.22917 PMC3562311

[FILIPESCUGAD290924C63] Quinlan AR, Hall IM. 2010 BEDTools: a flexible suite of utilities for comparing genomic features. Bioinformatics 26: 841–842. 20110278 10.1093/bioinformatics/btq033 PMC2832824

[FILIPESCUGAD290924C64] Régnier V, Vagnarelli P, Fukagawa T, Zerjal T, Burns E, Trouche D, Earnshaw W, Brown W. 2005 CENP-A is required for accurate chromosome segregation and sustained kinetochore association of BubR1. Mol Cell Biol 25: 3967–3981. 15870271 10.1128/MCB.25.10.3967-3981.2005 PMC1087704

[FILIPESCUGAD290924C65] Sanjana NE, Shalem O, Zhang F. 2014 Improved vectors and genome-wide libraries for CRISPR screening. Nat Methods 11: 783–784. 25075903 10.1038/nmeth.3047 PMC4486245

[FILIPESCUGAD290924C66] Santaguida S, Amon A. 2015 Short- and long-term effects of chromosome mis-segregation and aneuploidy. Nat Rev Mol Cell Biol 16: 473–485. 26204159 10.1038/nrm4025

[FILIPESCUGAD290924C67] Schwartzentruber J, Korshunov A, Liu X-Y, Jones DTW, Pfaff E, Jacob K, Sturm D, Fontebasso AM, Quang D-AK, Tönjes M, 2012 Driver mutations in histone H3.3 and chromatin remodelling genes in paediatric glioblastoma. Nature 482: 226–231. 22286061 10.1038/nature10833

[FILIPESCUGAD290924C68] Serrano M, Lin AW, McCurrach ME, Beach D, Lowe SW. 1997 Oncogenic *ras* provokes premature cell senescence associated with accumulation of p53 and p16INK4a. Cell 88: 593–602. 9054499 10.1016/s0092-8674(00)81902-9

[FILIPESCUGAD290924C69] Shelby RD, Vafa O, Sullivan KF. 1997 Assembly of CENP-A into centromeric chromatin requires a cooperative array of nucleosomal DNA contact sites. J Cell Biol 136: 501–513. 9024683 10.1083/jcb.136.3.501 PMC2134286

[FILIPESCUGAD290924C70] Shelby RD, Monier K, Sullivan KF. 2000 Chromatin assembly at kinetochores is uncoupled from DNA replication. J Cell Biol 151: 1113–1118. 11086012 10.1083/jcb.151.5.1113 PMC2174364

[FILIPESCUGAD290924C71] Shuaib M, Ouararhni K, Dimitrov S, Hamiche A. 2010 HJURP binds CENP-A via a highly conserved N-terminal domain and mediates its deposition at centromeres. Proc Natl Acad Sci 107: 1349–1354. 20080577 10.1073/pnas.0913709107 PMC2824361

[FILIPESCUGAD290924C72] Silva MCC, Bodor DL, Stellfox ME, Martins NMC, Hochegger H, Foltz DR, Jansen LET. 2012 Cdk activity couples epigenetic centromere inheritance to cell cycle progression. Dev Cell 22: 52–63. 22169070 10.1016/j.devcel.2011.10.014

[FILIPESCUGAD290924C73] Simeonova I, Jaber S, Draskovic I, Bardot B, Fang M, Bouarich-Bourimi R, Lejour V, Charbonnier L, Soudais C, Bourdon J-C, 2013 Mutant mice lacking the p53 C-terminal domain model telomere syndromes. Cell Rep 3: 2046–2058. 23770245 10.1016/j.celrep.2013.05.028

[FILIPESCUGAD290924C74] Sokolova M, Turunen M, Mortusewicz O, Kivioja T. 2017 Genome-wide screen of cell-cycle regulators in normal and tumor cells identifies a differential response to nucleosome depletion. Cell Cycle 16: 189–199. 27929715 10.1080/15384101.2016.1261765 PMC5283814

[FILIPESCUGAD290924C75] Stankovic A, Guo LY, Mata JF, Bodor DL, Cao X-J, Bailey AO, Shabanowitz J, Hunt DF, Garcia BA, Black BE, 2017 A dual inhibitory mechanism sufficient to maintain cell-cycle-restricted CENP-A assembly. Mol Cell 65: 231–246. 28017591 10.1016/j.molcel.2016.11.021 PMC5250512

[FILIPESCUGAD290924C76] Sturm D, Witt H, Hovestadt V, Khuong-Quang D-A, Jones DTW, Konermann C, Pfaff E, Tönjes M, Sill M, Bender S, 2012 Hotspot mutations in H3F3A and IDH1 define distinct epigenetic and biological subgroups of glioblastoma. Cancer Cell 22: 425–437. 23079654 10.1016/j.ccr.2012.08.024

[FILIPESCUGAD290924C77] Sun X, Clermont P-L, Jiao W, Helgason CD, Gout PW, Wang Y, Qu S. 2016 Elevated expression of the centromere protein-A (CENP-A)-encoding gene as a prognostic and predictive biomarker in human cancers. Int J Cancer 139: 899–907. 27062469 10.1002/ijc.30133

[FILIPESCUGAD290924C78] Talbert PB, Ahmad K, Almouzni G, Ausió J, Berger F, Bhalla PL, Bonner WM, Cande WZ, Chadwick BP, Chan SWL, 2012 A unified phylogeny-based nomenclature for histone variants. Epigenetics Chromatin 5: 7. 22650316 10.1186/1756-8935-5-7 PMC3380720

[FILIPESCUGAD290924C79] Tanaka K, Hirota T. 2009 Chromosome segregation machinery and cancer. Cancer Sci 100: 1158–1165. 19432891 10.1111/j.1349-7006.2009.01178.x PMC11158954

[FILIPESCUGAD290924C80] Tarailo-Graovac M, Chen N. 2009 Using RepeatMasker to identify repetitive elements in genomic sequences. Curr Protoc Bioinformatics 25: 4.10.1–4.10.14. 10.1002/0471250953.bi0410s25 19274634

[FILIPESCUGAD290924C81] Thompson SL, Bakhoum SF, Compton DA. 2010 Mechanisms of chromosomal instability. Curr Biol 20: R285–R295. 20334839 10.1016/j.cub.2010.01.034 PMC3781365

[FILIPESCUGAD290924C182] Tomonaga T. 2009 The kinetochore-cancer connection. In The kinetochore: from molecular discoveries to cancer therapy (ed. De Wulf P, Earnshaw WC), pp. 433–454. Springer, New York.

[FILIPESCUGAD290924C82] Tomonaga T, Matsushita K, Yamaguchi S, Oohashi T, Shimada H, Ochiai T, Yoda K, Nomura F. 2003 Overexpression and mistargeting of centromere protein-A in human primary colorectal cancer. Cancer Res 63: 3511–3516. 12839935

[FILIPESCUGAD290924C83] Valente V, Serafim RB, de Oliveira LC, Adorni FS, Torrieri R, Tirapelli DPDC, Espreafico EM, Oba-Shinjo SM, Marie SKN, Paçó-Larson ML, 2013 Modulation of HJURP (Holliday junction-recognizing protein) levels is correlated with glioblastoma cells survival. PLoS One 8: e62200. 23638004 10.1371/journal.pone.0062200 PMC3636219

[FILIPESCUGAD290924C84] Van Hooser AA, Ouspenski II, Gregson HC, Starr DA, Yen TJ, Goldberg ML, Yokomori K, Earnshaw WC, Sullivan KF, Brinkley BR. 2001 Specification of kinetochore-forming chromatin by the histone H3 variant CENP-A. J Cell Sci 114: 3529–3542. 11682612 10.1242/jcs.114.19.3529

[FILIPESCUGAD290924C85] Wang I-C, Chen Y-J, Hughes D, Petrovic V, Major ML, Park HJ, Tan Y, Ackerson T, Costa RH. 2005 Forkhead box M1 regulates the transcriptional network of genes essential for mitotic progression and genes encoding the SCF (Skp2–Cks1) ubiquitin ligase. Mol Cell Biol 25: 10875–10894. 16314512 10.1128/MCB.25.24.10875-10894.2005 PMC1316960

[FILIPESCUGAD290924C86] Wang Z, Zheng Y, Park HJ, Li J, Carr JR, Chen Y-J, Kiefer MM, Kopanja D, Bagchi S, Tyner AL, 2013 Targeting FoxM1 effectively retards p53-null lymphoma and sarcoma. Mol Cancer Ther 12: 759–767. 23427295 10.1158/1535-7163.MCT-12-0903 PMC3651795

[FILIPESCUGAD290924C87] Wonsey DR, Follettie MT. 2005 Loss of the forkhead transcription factor FoxM1 causes centrosome amplification and mitotic catastrophe. Cancer Res 65: 5181–5189. 15958562 10.1158/0008-5472.CAN-04-4059

[FILIPESCUGAD290924C88] Wu Q, Qian Y-M, Zhao X-L, Wang S-M, Feng X-J, Chen X-F, Zhang S-H. 2012 Expression and prognostic significance of centromere protein A in human lung adenocarcinoma. Lung Cancer 77: 407–414. 22542705 10.1016/j.lungcan.2012.04.007

[FILIPESCUGAD290924C89] Yau C, Meyer L, Benz S, Vaske C, Scott G, Egan B, Labhart P, Pourmand N, Benz CC. 2015 FOXM1 cistrome predicts breast cancer metastatic outcome better than FOXM1 expression levels or tumor proliferation index. Breast Cancer Res Treat 154: 23–32. 26456572 10.1007/s10549-015-3589-7

[FILIPESCUGAD290924C90] Zhang X, Cheng L, Minn K, Madan R, Godwin AK, Shridhar V, Chien J. 2014 Targeting of mutant p53-induced FoxM1 with thiostrepton induces cytotoxicity and enhances carboplatin sensitivity in cancer cells. Oncotarget 5: 11365–11380. 25426548 10.18632/oncotarget.2497 PMC4294351

[FILIPESCUGAD290924C91] Zhu J, Sammons MA, Donahue G, Dou Z, Vedadi M, Getlik M, Barsyte-Lovejoy D, Al-awar R, Katona BW, Shilatifard A, 2015 Gain-of-function p53 mutants co-opt chromatin pathways to drive cancer growth. Nature 525: 206–211. 26331536 10.1038/nature15251 PMC4568559

