# Supplementary material for: Essential role for centromeric factors following p53 loss and oncogenic transformation
Source: Genes Dev. 2017 Mar 1;31(5):463–80. doi: 10.1101/gad.290924.116 (PMC5393061; doi:10.1101/gad.290924.116)
Supplement: Supplemental Material [file supp_31_5_463__index.html]

Essential role for centromeric factors following p53 loss and oncogenic transformation — Supplemental Material 

# Essential role for centromeric factors following p53 loss and oncogenic transformation

## Supplemental Material

- Supplemental\_FigS1.pdf
- Supplemental\_FigS2.pdf
- Supplemental\_FigS3.pdf
- Supplemental\_FigS4.pdf
- Supplemental\_FigS5.pdf
- Supplemental\_FigS7.pdf
- Supplemental\_Table1.pdf
- Supplemental\_FigS6.pdf
