# Supplementary material for: Essential role for centromeric factors following p53 loss and oncogenic transformation
Source: Genes Dev. 2017 Mar 1;31(5):463–80. doi: 10.1101/gad.290924.116 (PMC5393061; doi:10.1101/gad.290924.116)
Supplement: Supplemental Material [file supp_gad.290924.116_Supplemental_FigS1.pdf]

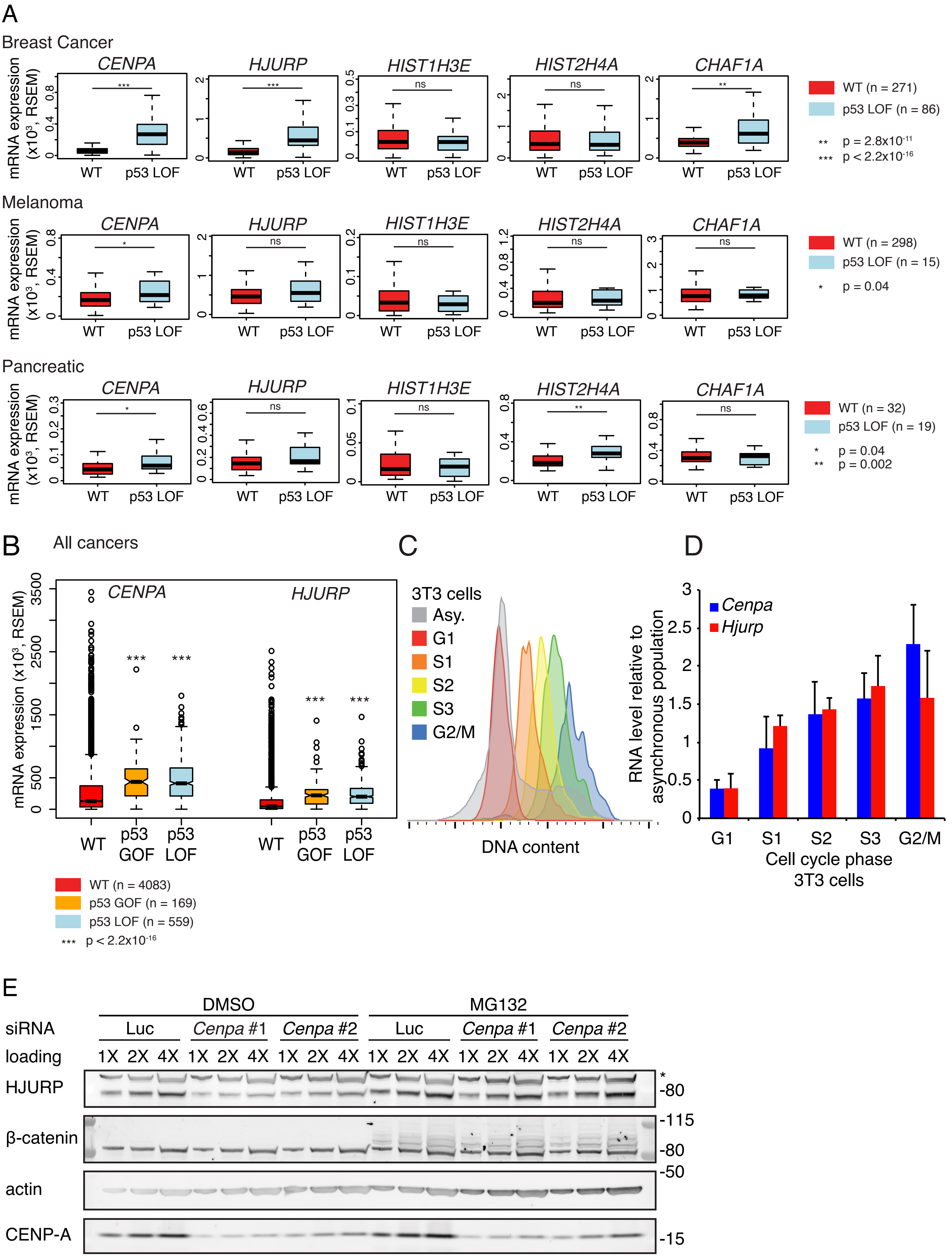
A
Breast Cancer
CENPA
HJURP
HIST1H3E
HIST2H4A
CHAF1A
mRNA expression (x10³, RSEM)
WT
p53 LOF
WT (n = 271)
p53 LOF (n = 86)
** p = 2.8x10-11
*** p < 2.2x10-16
Melanoma
WT (n = 298)
p53 LOF (n = 15)
* p = 0.04
Pancreatic
WT (n = 32)
p53 LOF (n = 19)
* p = 0.04
** p = 0.002
B
All cancers
WT
p53 GOF
p53 LOF
WT (n = 4083)
p53 GOF (n = 169)
p53 LOF (n = 559)
*** p < 2.2x10-16
C
3T3 cells
Asy.
G1
S1
S2
S3
G2/M
DNA content
D
Cenpa
Hjurp
RNA level relative to asynchronous population
Cell cycle phase
3T3 cells
E
DMSO
MG132
siRNA
Luc
Cenpa #1
Cenpa #2
loading
1X 2X 4X
HJURP
β-catenin
actin
CENP-A
-80
-115
-50
-15

Supplemental Figure S1 related to Figure 1

(A) Box plot comparisons of relative expression (mRNA) of genes coding for CENP-A (*CENPA*), HJURP (*HJURP*), CAF-1 p150 (*CHAF1A*), H3.1 (*HIST1H3E*), and H4 (*HIST2H4A*), from breast (top), melanoma (middle), and pancreatic (bottom) cancers classified according to p53 status (TCGA data). Tumors are either WT for TP53, (diploid with no mutations) or TP53 loss-of-function (LOF) (homozygous deletion or heterozygous deletion + TP53 mutation featuring nonsense or in frame truncations resulting in p53 loss). All other TP53 mutants were excluded. mRNA levels are expressed in RSEM units. We used Wilcoxon rank sum tests to compute significance.

(B) Box plot comparison of relative expression (mRNA) of genes coding for CENP-A and HJURP as in (A), for all cancers. We compare WT and p53 LOF tumors to p53 gain-of-function (GOF) single missense mutations (R175H, R248Q, R248W, R249S, R273H).

(C) Cell cycle profiles of NIH-3T3 cells sorted according to DNA content, measured by Hoechst 33342 incorporation. Profiles of the starting (Asy = asynchronous) population, and 5 isolated fractions for each cell cycle phase are shown.

(D) RT-qPCR analysis of Hjurp and Cenpa mRNA levels in NIH-3T3 cell sorted populations shown in (B). Expression normalized to Gapdh and Idh mRNA and set to 1 for the asynchronous population. Error bars represent the standard error of at least 3 experiments.

(E) CENP-A depletion leads to proteosome-dependent degradation of HJURP. Western blot of RIPA- soluble extracts of NIH-3T3 cells transfected for 48 hours with Control siRNA or two siRNAs against Cenpa (Cenpa #1 and Cenpa #2). Prior to harvest, cells were treated for 4 hours with DMSO alone or the protease inhibitor MG132. * marks a non-specific band detected with the HJURP antibody. We measured β-catenin levels as a control for efficient inhibition of the proteasome. γ tubulin is used as a loading control. A two-fold dilution series of each extract is represented by 4X, 2X, 1X. Molecular weight protein markers are indicated on the right.
