# Supplementary material for: Essential role for centromeric factors following p53 loss and oncogenic transformation
Source: Genes Dev. 2017 Mar 1;31(5):463–80. doi: 10.1101/gad.290924.116 (PMC5393061; doi:10.1101/gad.290924.116)
Supplement: Supplemental Material [file supp_gad.290924.116_Supplemental_FigS2.pdf]

A

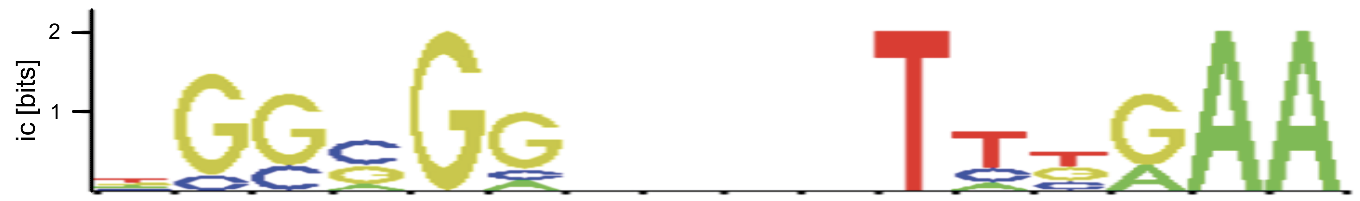


| | 1 | 2 | 3 | 4 | 5 | 6 | 7 | 8 | 9 | 10 | 11 | 12 | 13 | 14 | 15 | 16 |
|---|---|---|---|---|---|---|---|---|---|---|---|---|---|---|---|---|
| A [ | 0.25 | 0 | 0 | 0.125 | 0 | 0.125 | 0.25 | 0.25 | 0.25 | 0.25 | 0 | 0.125 | 0 | 0.25 | 1 | 1 ] |
| C [ | 0.125 | 0.125 | 0.25 | 0.5 | 0 | 0.125 | 0.25 | 0.25 | 0.25 | 0.25 | 0 | 0.25 | 0.25 | 0 | 0 | 0 ] |
| G [ | 0.25 | 0.875 | 0.75 | 0.375 | 1 | 0.75 | 0.25 | 0.25 | 0.25 | 0.25 | 0 | 0 | 0.375 | 0.75 | 0 | 0 ] |
| T [ | 0.375 | 0 | 0 | 0 | 0 | 0 | 0.25 | 0.25 | 0.25 | 0.25 | 1 | 0.625 | 0.375 | 0 | 0 | 0 ] |

| | CDE (4bp) CHR | PFM Score |
|---|---|---|
| B-myb | TGGCGGGAGATAGGAA | 10.27 |
| Cks1 | GGGCGGTGTGTTTGAA | 11.17 |
| Cyclin B2 | CGGCGCGGTATTTGAA | 9.34 |
| Fancd2 | AGCGGGAAAGTCGAAA | 8.10 |
| Fanci | AGCGGGAATTTTGGAA | 9.91 |
| Fancr | TGGCGGGATTTCCGAA | 10.36 |
| Plk4 | GCGGGAATTTTTCAAA | 6.59 |
| Tome-1 | TGGAGGGAAGTTTGAA | 10.49 |

B

| | | |
|---|---|---|
| *Cenpa* (+115) | CGGGTGATTTTTTGAA | 8.17 |
| *Hjurp* (+97) | GGGCGCCACGTTTGAT | 7.44 |

C

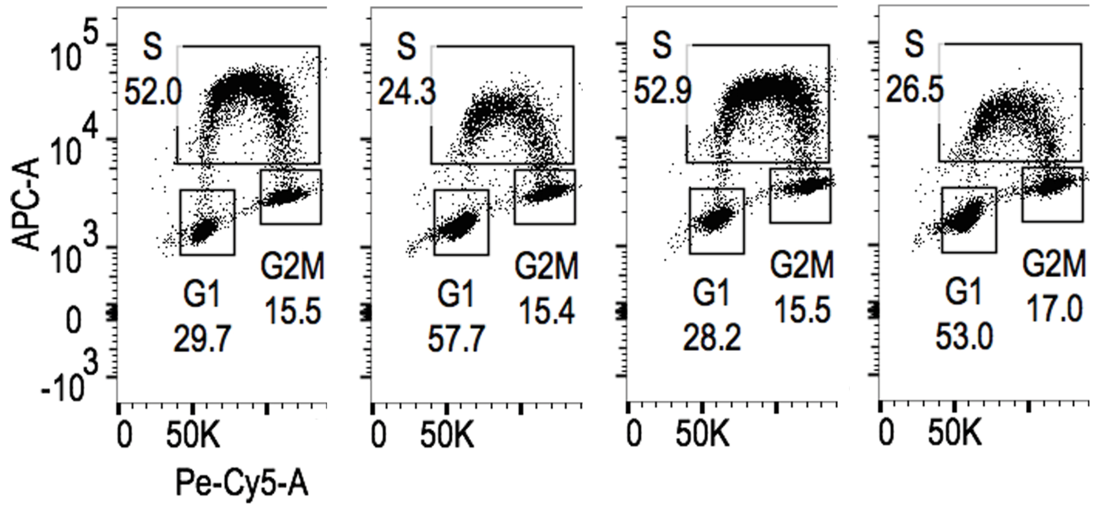


| | | | | |
|---|---|---|---|---|
| Luciferase plasmid | Hjurp WT | Hjurp WT | Hjurp CDE Mut | Hjurp CDE Mut |
| Nutlin | - | + | - | + |

**Supplemental Figure S2 related to Figure 2**

(A) Positional frequency matrix (PFM) used to search for candidate CDE/CHR motifs *in silico* in gene promoters. The sequence of 8 functional CDE/CHR motifs (from the mouse genes *B-myb*, *Cks1*, *Cyclin B2*, *Fancd2*, *Fanci*, *Fancr*, *Plk4* and *Tome-1* (Jaber et al., 2016) were used to define the PFM, and the 8 motifs were then evaluated with the PFM. Scores from 6.59 (*Plk4*) to 11.17 (*Cks1*) were obtained.

(B) The same PFM was used to analyze sequences at the *Cenpa* and *Hjurp* promoters. Candidate CDE/CHR motifs were found close to the transcription start site (TSS) of each gene, with PFM scores of 8.17 (*Cenpa*) and 7.44 (*Hjurp*). For *Cenpa* the CHR element perfectly matches the consensus sequence, whereas the CDE element perfectly matches the consensus sequence for *Hjurp.* These elements were mutated in luciferase assays (see Figure 2D-E). Numbers in parentheses are positions relative to the TSS.

(C) Cell cycle analysis (Edu/PI staining) by flow cytometry in NIH/3T3 cells transfected with a WT or mutant Hjurp Luciferase reporter plasmid (see Figure 2E), untreated or treated with 10 $\mu$M nutlin for 24 hr.
