# Supplementary material for: Essential role for centromeric factors following p53 loss and oncogenic transformation
Source: Genes Dev. 2017 Mar 1;31(5):463–80. doi: 10.1101/gad.290924.116 (PMC5393061; doi:10.1101/gad.290924.116)
Supplement: Supplemental Material [file supp_gad.290924.116_Supplemental_FigS3.pdf]

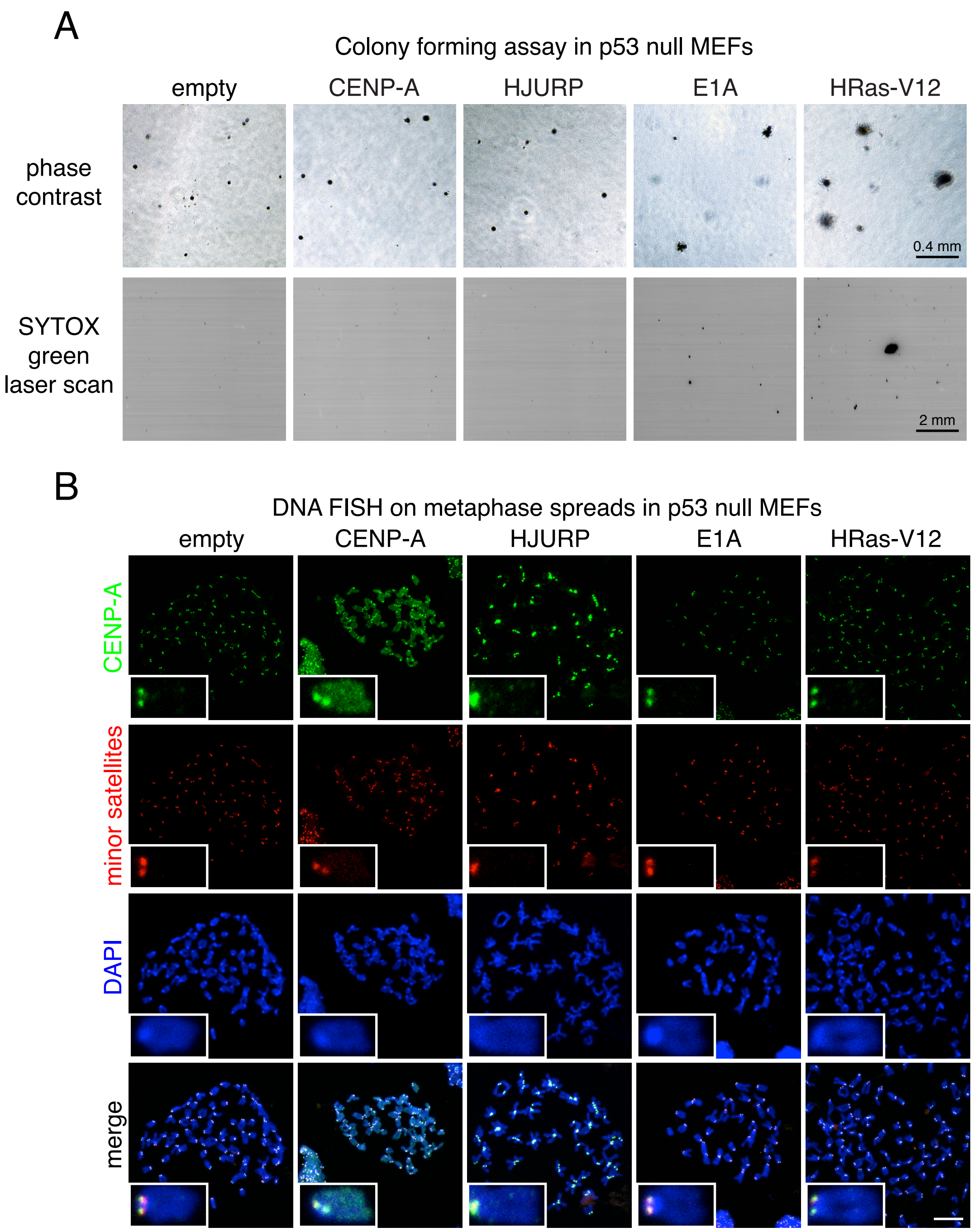


**Supplemental Figure S3 related to Figure 3**

(A) Soft agar colony forming assay in p53 null MEFs transduced with the indicated retroviral construct. We stained colonies with Sytox Green 4 weeks after seeding. (B) DNA FISH on metaphase spreads of p53 null MEFs transduced with the indicated retroviral construct. We stained cells with antibodies for CENP-A, LNA FISH probes for minor satellites (site of centromeric CENP-A deposition), and DAPI. Insets show individual magnified chromosomes. Scale bar, 10 $\mu$m.
