# Supplementary material for: Essential role for centromeric factors following p53 loss and oncogenic transformation
Source: Genes Dev. 2017 Mar 1;31(5):463–80. doi: 10.1101/gad.290924.116 (PMC5393061; doi:10.1101/gad.290924.116)
Supplement: Supplemental Material [file supp_gad.290924.116_Supplemental_FigS4.pdf]

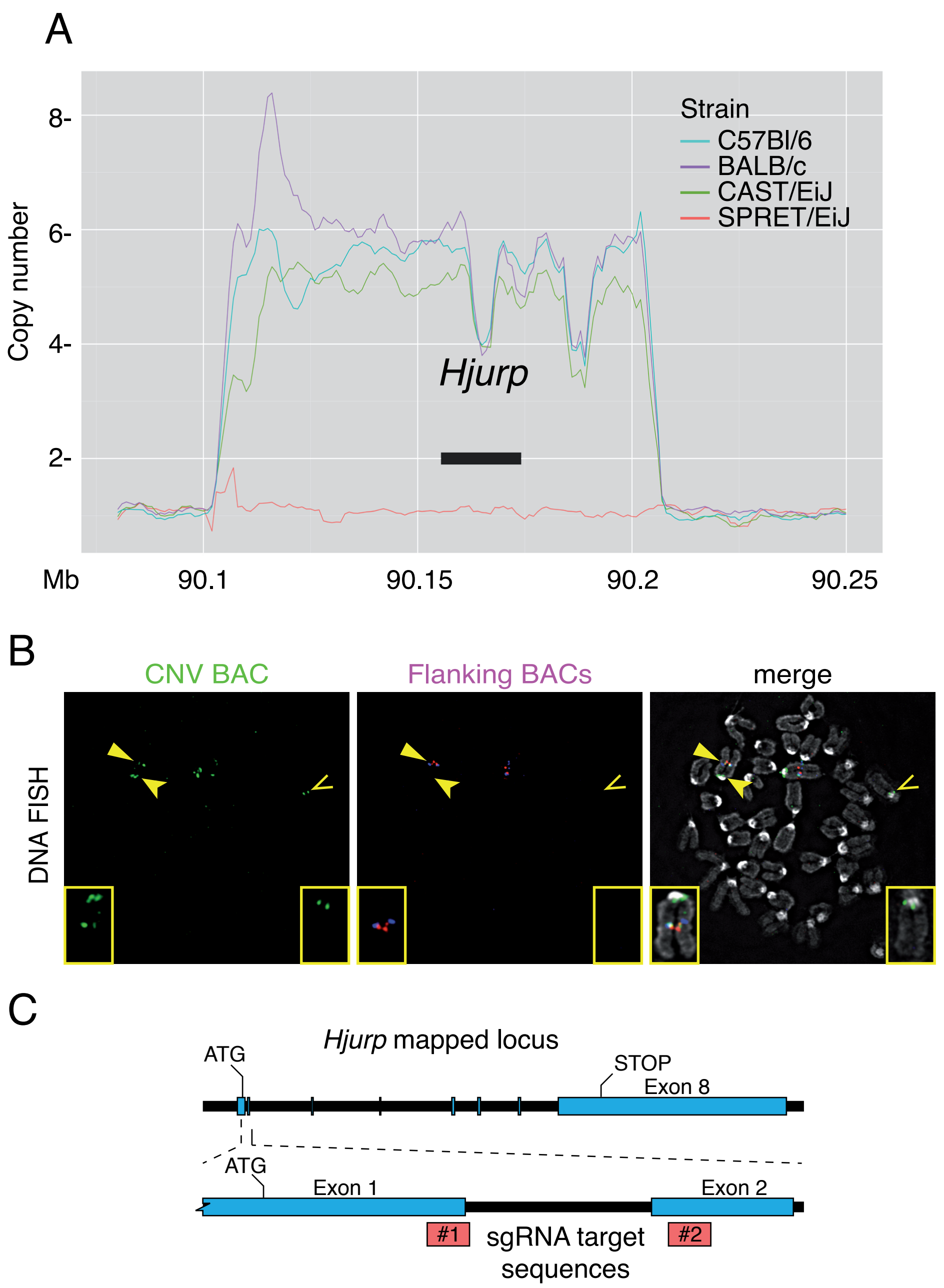

Supplemental Figure S4 related to Figure 4

(A) Copy number variation (CNV) from mouse paired-end genome resequencing data in C57BL/6, BALB/c, CAST/EiJ, and SPRET/EiJ mice. A ~100kb region on chromosome 1 comprising the Hjurp locus (black bar) is shown. Outside this region, the copy number of 1 corresponds to two alleles.

(B) DNA FISH on metaphase spreads prepared from activated lymphocytes of wild-type C57BL/6 mice. The green probe corresponds to a BAC covering the region in chromosome 1 shown in (A), harboring the CNV (CNV BAC). The flanking BACs represent probes for single-copy regions up- and downstream of the mapped region in chromosome 1. The triangles indicate the mapped *Hjurp* locus on chromosome 1qD (left insert), the closed arrowhead indicates *Hjurp* paralogues outside of the mapped locus, and the open arrowhead indicates the centromeric region of another chromosome (right inset).

(C) Structure of the *Hjurp* mapped locus according to the mm9 and mm10 mouse genome assemblies. In the zoomed region, the red boxes (21 nt) depict the recognition sites of the two distinct sgRNA constructs used in combination with Cas9 to knock out *Hjurp*.

D

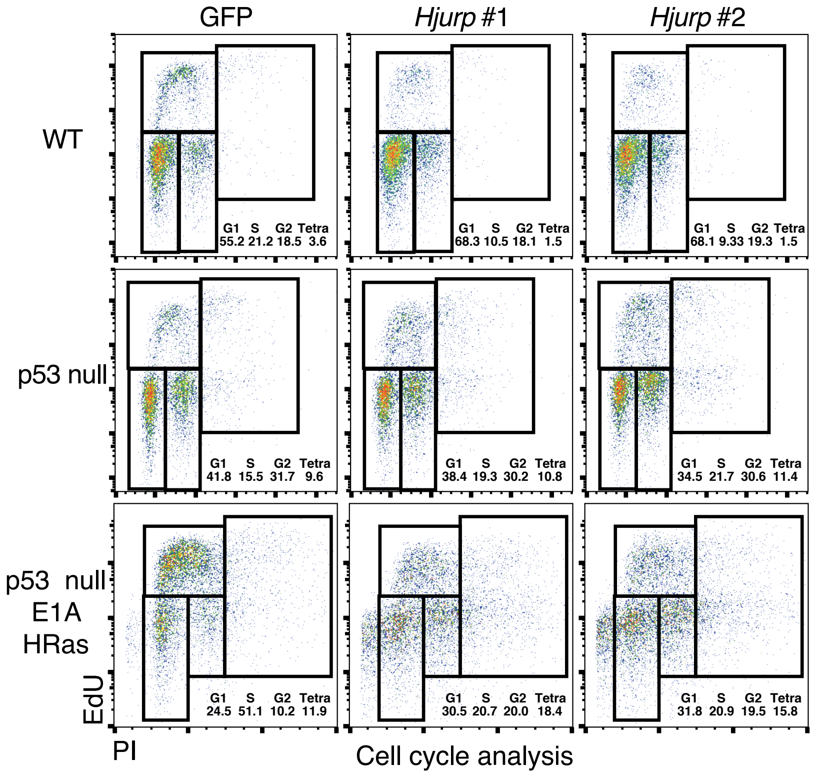

E

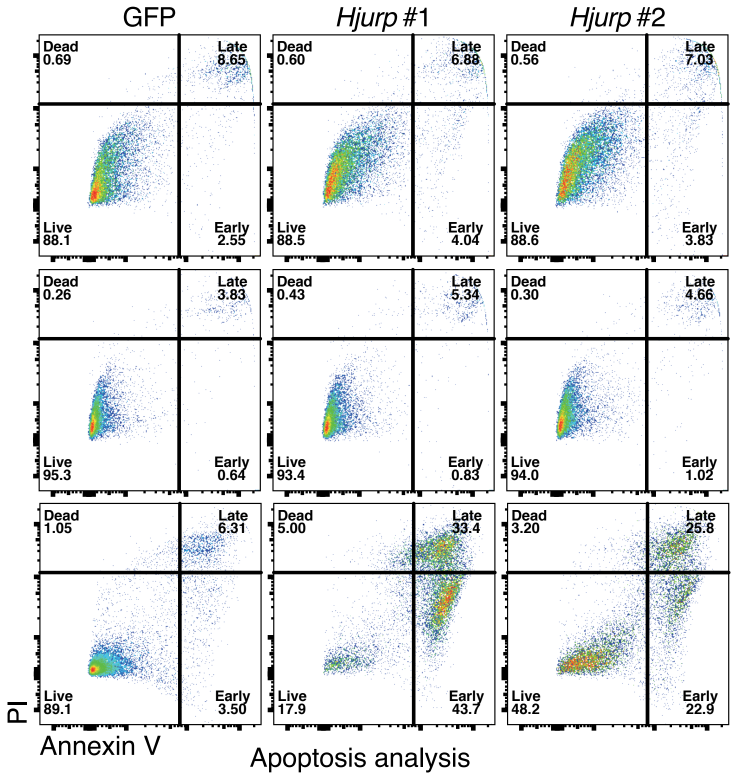

Supplemental Figure S4 related to Figure 4

(D) Cell cycle analysis by flow cytometry (Edu and PI staining) in a representative experiment in MEFs following *Hjurp* KO at day 6 post transduction with CRISPR constructs, following puromycin selection.

(E) Apoptosis analysis by flow cytometry (Annexin V and PI staining) in a representative experiment in MEFs following *Hjurp* KO at day 6 post transduction with CRISPR constructs, following puromycin selection.

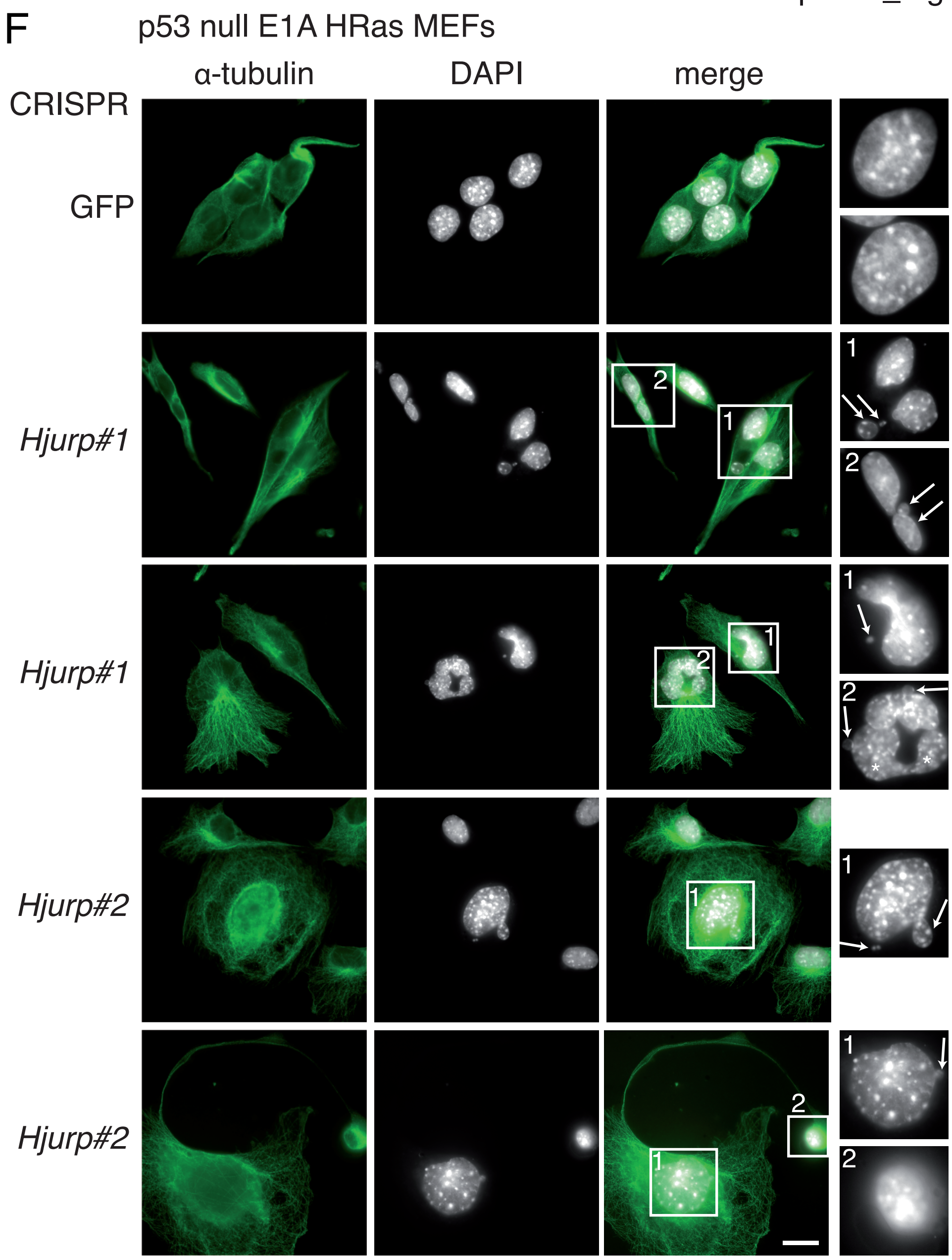

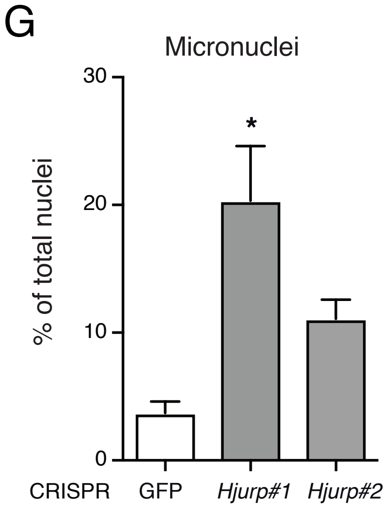

Supplemental Figure 4 relating to Figure 4

(F) Immunofluoresence images of p53 null E1A HRas-V12-transformed MEFs at day 6 post-transduction with CRISPR constructs, following puromycin selection. One representative image for GFP CRISPR, and two representative images for each Hjurp CRISPR (#1 and #2) are shown. We stained cells with antibodies for α-tubulin and DAPI. Insets show individual magnified nuclei. Micronuclei are highlighted by arrows. Scale bar, 10 μm.
