# Supplementary material for: Essential role for centromeric factors following p53 loss and oncogenic transformation
Source: Genes Dev. 2017 Mar 1;31(5):463–80. doi: 10.1101/gad.290924.116 (PMC5393061; doi:10.1101/gad.290924.116)
Supplement: Supplemental Material [file supp_gad.290924.116_Supplemental_FigS5.pdf]

Filipescu_Fig S5

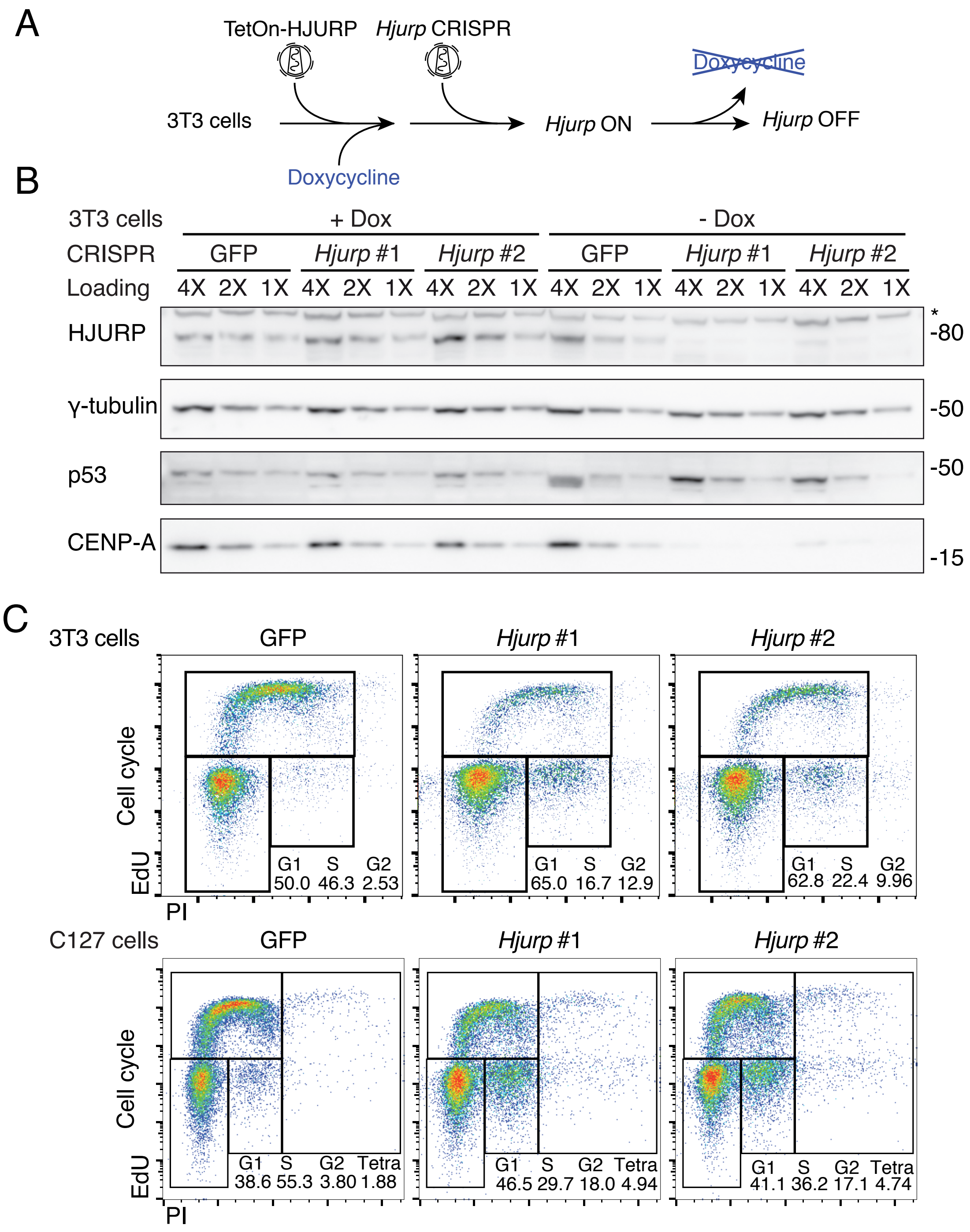

Supplemental Figure 5 relating to Figure 5

(A) Scheme outlining inducible HJURP rescue experiment in NIH-3T3 cells. We transduced cells with a Dox-inducible (Tet-On) CRISPR-resistant Hjurp transgene. In the presence of Dox (Hjurp On), we subsequently transduced cells with CRISPR-lentiviral particles against GFP (control) or 2 sgRNA constructs targeting Hjurp. Doxycycline withdrawal switches off the HJURP transgene (Hjurp Off)

(B) Western blot in RIPA-soluble extracts of NIH-3T3 cells, 10 days after transduction of CRISPR lentiviral particles, and puromycin selection in the presence of Dox (+Dox, Hjurp On) or 6 days following Dox withdrawal (-Dox, Hjurp Off). * marks a non-specific band detected with the HJURP antibody. γ tubulin is used as a loading control. A two-fold dilution series of each extract is represented by 4X, 2X, 1X. Molecular weight markers are indicated on the right.

(C) Cell cycle (Edu/PI) analysis by flow cytometry in NIH-3T3 cells and C127 cells, 14 days and 6 days, respectively, post transduction of CRISPR lentiviral particles and following puromycin selection.
