# Supplementary material for: Essential role for centromeric factors following p53 loss and oncogenic transformation
Source: Genes Dev. 2017 Mar 1;31(5):463–80. doi: 10.1101/gad.290924.116 (PMC5393061; doi:10.1101/gad.290924.116)
Supplement: Supplemental Material [file supp_gad.290924.116_Supplemental_FigS7.pdf]

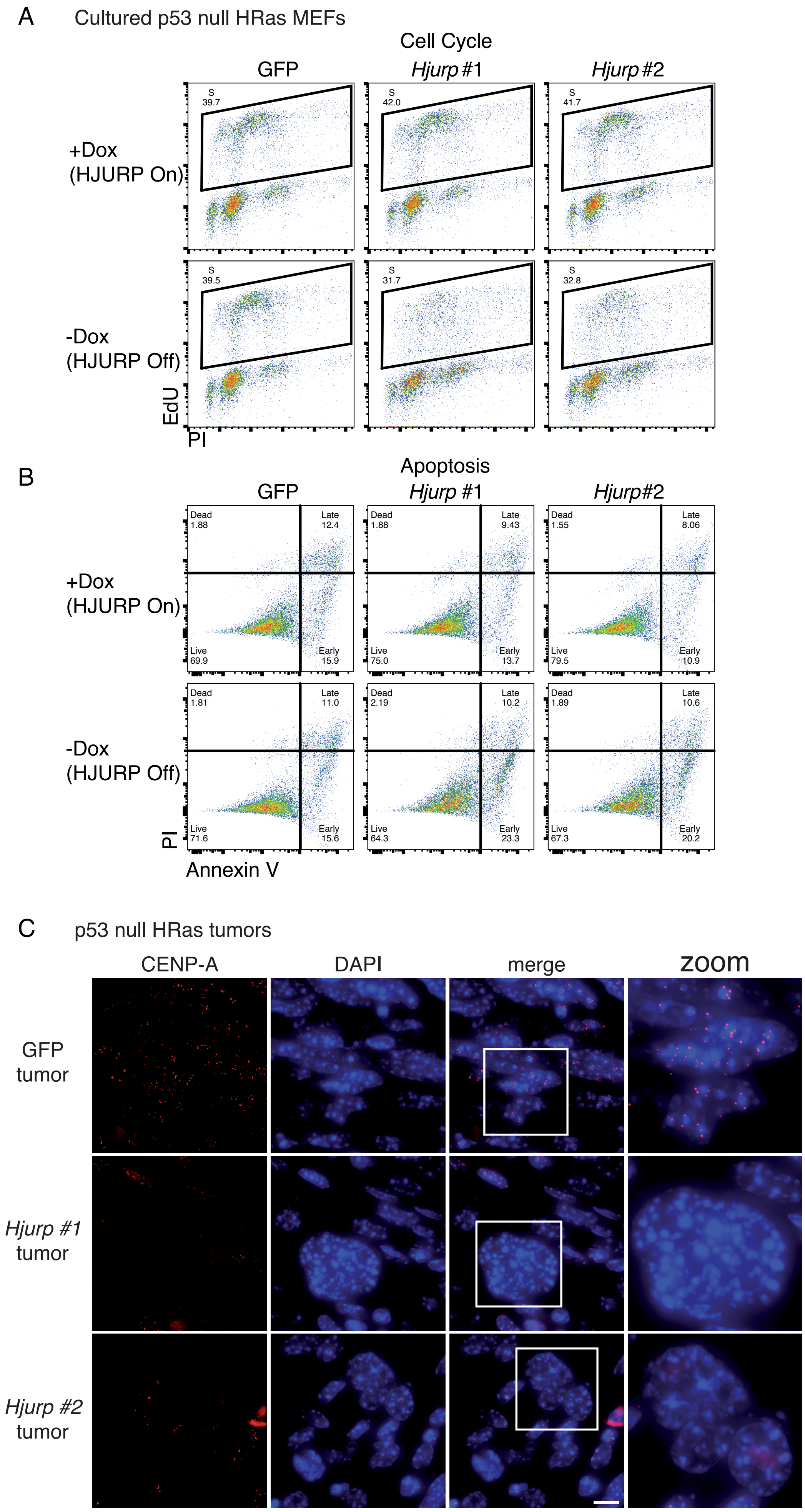
A
Cultured p53 null HRas MEFs
Cell Cycle
GFP
Hjurp #1
Hjurp #2
+Dox
(HJURP On)
-Dox
(HJURP Off)
S 39.7
S 42.0
S 41.7
S 39.5
S 31.7
S 32.8
EdU
PI
B
Apoptosis
GFP
Hjurp #1
Hjurp#2
+Dox
(HJURP On)
-Dox
(HJURP Off)
Dead 1.88
Late 12.4
Live 69.9
Early 15.9
Dead 1.88
Late 9.43
Live 75.0
Early 13.7
Dead 1.55
Late 8.06
Live 79.5
Early 10.9
Dead 1.81
Late 11.0
Live 71.6
Early 15.6
Dead 2.19
Late 10.2
Live 64.3
Early 23.3
Dead 1.89
Late 10.6
Live 67.3
Early 20.2
PI
Annexin V
C
p53 null HRas tumors
CENP-A
DAPI
merge
zoom
GFP tumor
Hjurp #1 tumor
Hjurp #2 tumor

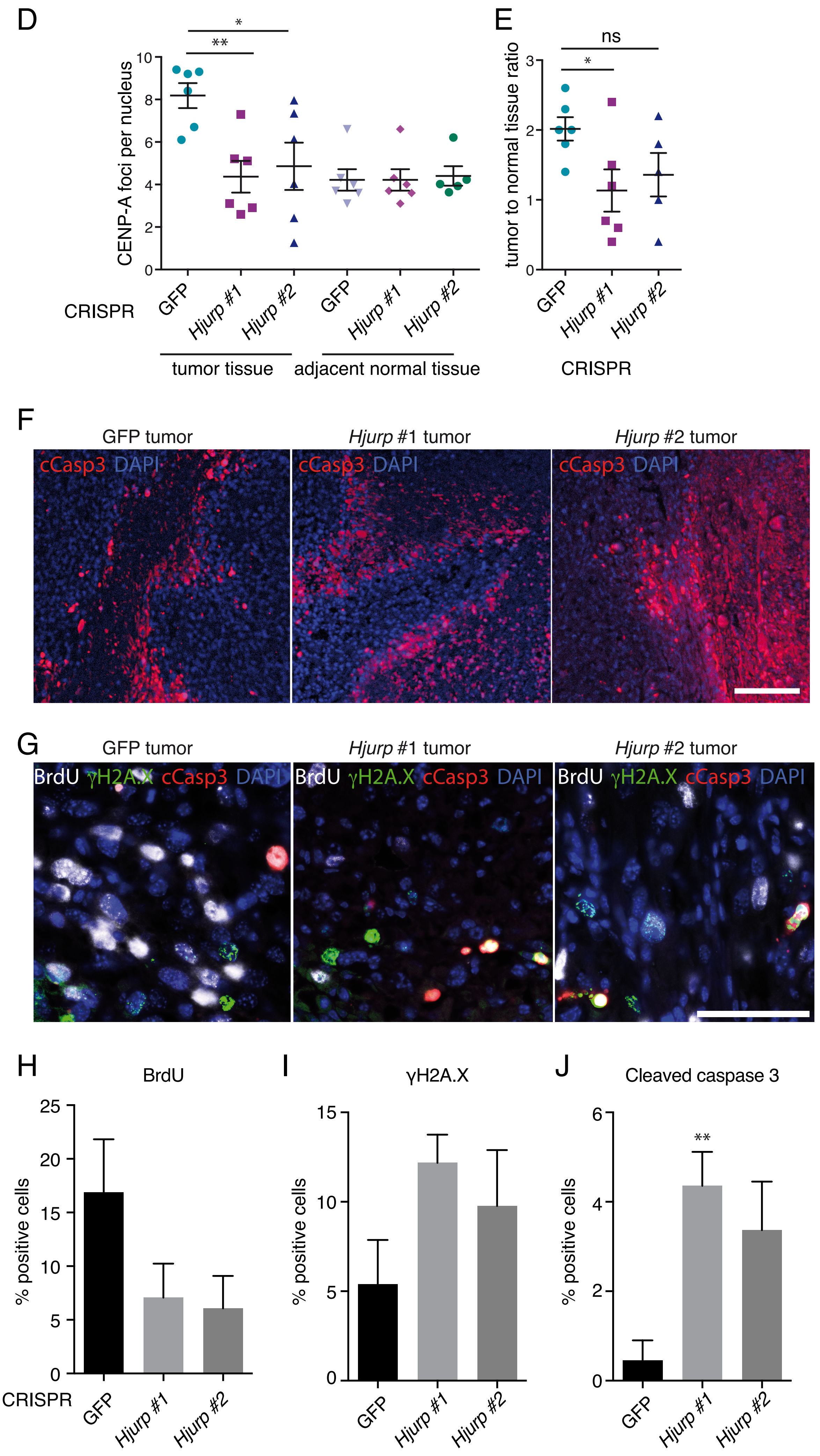
D
E
F
G
H
I
J
CENP-A foci per nucleus
tumor to normal tissue ratio
CRISPR
GFP
Hjurp #1
Hjurp #2
tumor tissue
adjacent normal tissue
ns
GFP tumor
Hjurp #1 tumor
Hjurp #2 tumor
cCasp3 DAPI
BrdU γH2A.X cCasp3 DAPI
BrdU
γH2A.X
Cleaved caspase 3
% positive cells

Supplemental Figure S7 related to Figure 7

(A) Cell cycle (Edu/PI staining) and (B) apoptosis analysis (annexin V/PI staining) by flow cytometry in p53 null, HRas-transformed MEFs, expressing CRISPR-resistant inducible Hjurp transgene (TET-On), following CRISPR lentiviral transduction, either in the presence of Dox (Hjurp On) or 8 days following Dox withdrawal (Hjurp Off). (C) Immunofluorescence images of tumor tissue from allograft assays. Tumor sections were stained with antibodies for CENP-A and DAPI. Images are Z-stack projections. Right panel is a zoom of the merged image. Scale bar 10 μm. (D) Quantification of the number of CENP-A foci per nucleus on single plane images, performed in 6 tumors per CRISPR condition, with at least 25 cells counted per tumor. We also performed quantification of CENP-A foci in adjacent normal tissue, which we defined by the presence of skin follicles (T-test, * $p < 0.05$, ** $p < 0.005$). (E) Data in (C) represented as a ratio of number of CENP-A foci in tumor/ number of CENP-A foci in adjacent normal tissue (T-test, * $p < 0.05$). (F) and (G) Immunofluorescence images of tumor tissue from allograft assays. (F) Tumor sections were stained with antibodies for cleaved caspase-3 and DAPI. Scale bar 100 µm. (G) BrdU was injected in mice intraperitoneally prior to tumor harvest. Tumor sections were stained with antibodies for Brdu, γH2A.X, cleaved caspase-3, and DAPI. Scale bar 50 µm. (H) Quantification of the number cells positive for BrdU, or (I) γH2A.X or (J) cleaved caspase-3. Bars represent percentage of positive cells +/- standard error of the mean of 3 counted fields (T-test, ** $p < 0.005$). 200-500 cells counted in each instance.
