# Supplementary material for: Essential role for centromeric factors following p53 loss and oncogenic transformation
Source: Genes Dev. 2017 Mar 1;31(5):463–80. doi: 10.1101/gad.290924.116 (PMC5393061; doi:10.1101/gad.290924.116)
Supplement: Supplemental Material [file supp_gad.290924.116_Supplemental_Table1.pdf]

Mouse Gene	Forward Primer (5'-3')	Reverse Primer (5'-3')
Cenpa	CTCCAGTGTAGGCTCTCAGAC	CTGAAAGGCTTCTTCCTGAACA
Hjurp	CTCTGGATGCCAGGATGC	CCACAATGGCCATATCAGC
Gapdh	TGCACCACCAACTGCTTAGC	GGCATGGACTGTGGTCATGAG
Idh	AGAAAATGTGGAAGAGCCCTAACG	TGCCAGCTCGATCTACCACAAAAT

Supplementary Table 1. DNA sequences for primers used in RT-qPCR experiments in Figure 1.
The primers for *Gapdh* and *Idh* were used for normalization.

Mouse Gene	Forward Primer (5'-3')	Reverse Primer (5'-3')
Arp	ATCTGCTGCATCTGCTTG	CGACCTGGAAGTCCAACTAC
Cenpa	CCGTGGTGTGGATTTTTGGT	GCTTCTGCTGCCTCCTGAAG
Hjurp	GGCAAGCTCCAGAAGGTGATT	GCCAGTTCTCCTCTAAGCTCGTA
Ppia	CAGTGCTCAGAGCTCGAAAGTTT	TCTCCTTCGAGCTGTTTGCA
**Human Gene**		
CENPA	TGGACTTCAATTGGCAAGCC	AGTAACTCGGCCTGCATGTA
HJURP	CACGTCAGACCAGGAAGAGT	TTCCAGCTCTGTTACCTGCA
PPIA	CAAATGCTGGACCCAACACA	TGCCATCCAACCACTCAGTCT
RPLP0	CTTGTCTGTGGAGACGGATTACAC	TACGCCAAGAAGGCCTTGA

Supplementary Table 2. DNA sequences for primers used in RT-qPCR experiments in Figure 2.
The primers for *Arp* and *Ppia* were used for normalization in mouse samples, and the primers for *PPIA* and *RPLP0* were used for normalization in human samples.
