# Supplementary material for: Essential role for centromeric factors following p53 loss and oncogenic transformation
Source: Genes Dev. 2017 Mar 1;31(5):463–80. doi: 10.1101/gad.290924.116 (PMC5393061; doi:10.1101/gad.290924.116)
Supplement: Supplemental Material [file supp_gad.290924.116_Supplemental_FigS6.pdf]

Filipescu_Fig S6

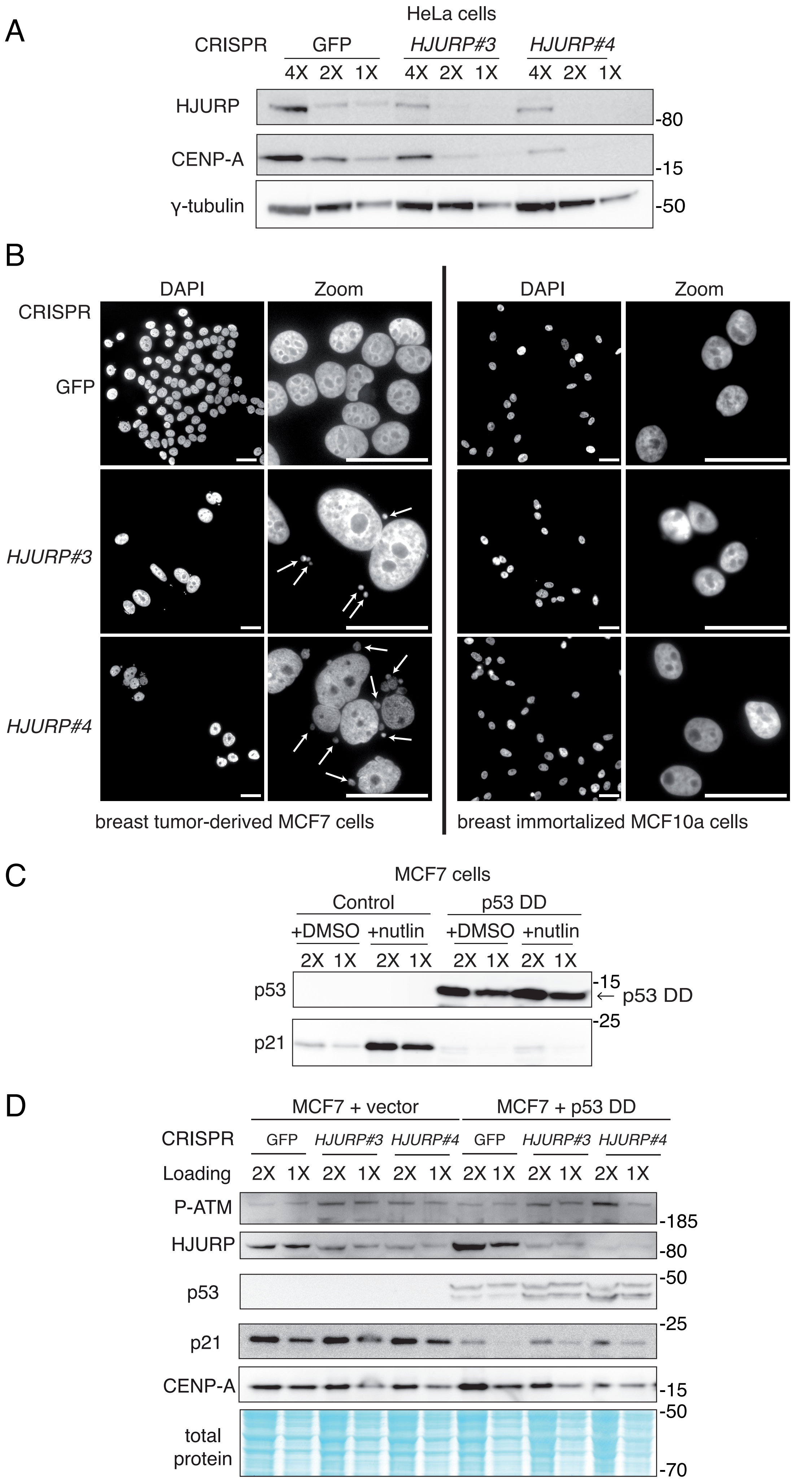

Supplemental Figure 6 relating to Figure 6

(A) Western blot in RIPA-soluble extracts of Hela cells of HJURP and CENP-A levels, 6 days after lentiviral transduction of CRISPR constructs against GFP (Control) or two sgRNA constructs targeting *HJURP* (*HJURP#3* and *HJURP#4*), following puromycin selection. γ-tubulin is used as a loading control. A two-fold dilution series of each extract is represented by 4X, 2X, 1X. Molecular weight markers are shown on the right.

(B) Immunofluoresence images of MCF7 or MCF10a cells, at day 6 post-transduction with CRISPR constructs, following puromycin selection. We stained cells with DAPI. Micronuclei are highlighted by arrows. Scale bar, 10 μm.

(C) Western blot in RIPA-soluble extracts of MCF7 cells transduced with a control vector (MCF7 + vector) or with a p53 dominant negative construct which abrogates p53 function (p53 DD), untreated or treated with 10 μM Nutlin for 24 hr. We confirmed expression of the p53 DD mutant (highlighted by arrow), and loss of p21 activation upon nutlin treatment in cells expressing p53 DD mutant. A two-fold dilution series of each extract is represented by 2X, 1X. Molecular weight markers are shown on the right.

(D) Western blot of HJURP and CENP-A levels in MCF7 cells expressing either control vector (MCF7 + vector) or p53 dominant negative mutant (p53 DD), 6 days after transduction with CRISPR-lentiviral particles against GFP (control) or 2 sgRNA constructs targeting *HJURP* (*HJURP#3* and *HJURP#4*), following puromycin selection. We also examined phospho-ATM and p53 levels. We detected total protein with Memcode protein stain. A two-fold dilution series of each extract is represented by 4X, 2X, 1X. Molecular weight markers are shown on the right.
